# Molecular Hydrogen Production from Formic Acid by Cationic Phenanthroline Ruthenium Complexes: Experimental and DFT Mechanistic Insights

**DOI:** 10.1021/acsomega.5c10186

**Published:** 2026-01-21

**Authors:** Gustavo H. C. Masson, Douglas H. N. Santos, Lucas S. Santos, André L. Bogado, Leonardo T. Ueno, Beatriz E. Goi, Walter Baratta, Valdemiro P. Carvalho-Jr

**Affiliations:** † 28108 Faculdade de Ciências e Tecnologia (FCT) da Universidade Estadual Paulista (UNESP), Presidente Prudente, São Paulo 19060-900, Brazil; ‡ Dipartimento di Scienze AgroAlimentari, Ambientali e Animali (DI4A)—Università di Udine Via Cotonificio108, 33100 Udine, Italy; § Instituto de Química, 28132 Universidade Estadual de Campinas (UNICAMP), 13083-970 Campinas, São Paulo, Brazil; ∥ Instituto de Ciências Exatas e Naturais Do Pontal, 28119 Universidade Federal de Uberlândia, ICENP−UFU, 38304-402 Ituiutaba, Minas GeraisG, Brazil; ⊥ Comando-Geral de Tecnologia Aeroespacial, Departamento de Química, Instituto Tecnológico de Aeronáutica (ITA), São José Dos Campos, São Paulo 12228-900, Brazil

## Abstract

A series of new monocationic Ru complexes containing phenanthroline derivatives were developed. The monometallic complexes [Ru­(κ^2^-OAc)­(dppb)­(N,N)]­OAc derivatives were synthesized in high yield via the reaction between [Ru­(κ^2^-OAc)_2_dppb] and the corresponding N,N ligand. Additionally, dinuclear [(dppb)­(κ^2^-OAc)­(Ru­(μ-*N*,*N*--C,N)­Ru­(κ^2^-OAc)­(dppb)]­OAc complexes were synthesized from equimolar amounts of the appropriate monometallic complex and [Ru­(κ^2^-OAc)_2_dppb]. All complexes were characterized by NMR, FTIR, UV–vis spectroscopy, and cyclic voltammetry. These precatalysts display selective catalytic activity toward dehydrogenation of formic acid for H_2_ production, with the dinuclear systems demonstrating superior performance, achieving up to 100% conversion under optimized conditions. The dinuclear system maintained consistent TOF_50_ values through several catalytic cycles, demonstrating excellent stability. Mechanism investigations revealed the formation of two Ru-monohydride species, showing a *fac*-RuHP_2_ and a *mer*-RuHP_2_ arrangement, respectively, formed via substitution of a κ^2^-OAc by a κ^2^-O_2_CH followed by a β-elimination, where both are involved in the mechanisms. DFT calculations of the species involved in the mechanism showed that *fac*-RuHP_2_ is lower in energy than *mer*-RuHP_2_. The complexes were additionally applied in the transfer hydrogenation of CO_2_ to produce formic acid with 2-propanol.

## Graphical abstract

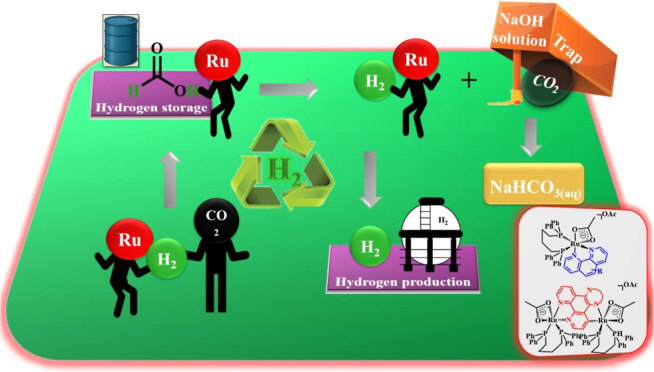

## 1 Introduction

Global energy demand continues to grow despite declining birth rates, with fossil fuels remaining the dominant source. However, these energy sources are limited in availability and cannot sustain a long-term global demand. Moreover, CO_2_ emissions from fossil fuel combustion exacerbate global warming, imposing significant economic and environmental consequences. The excessive emission of CO_2_ has caused serious problems, including ocean acidification, biodiversity loss, and rising sea levels.^1^ One of the most promising strategies to address these challenges is transitioning to cleaner energy sources, particularly H_2_.^2^

In terms of energy per unit of mass, H_2_ is more energy-dense than conventional hydrocarbon fuels. However, considering the challenges and safety risks associated with hydrogen storage and transportation, the use of liquid organic hydrogen carriers (LOHCs) offers considerable advantages, addressing many issues inherent in conventional H_2_ storage methods.^3,4^ Although formic acid (FA) has a relatively lower hydrogen weight percentage (4.4% wt) compared to some alternative hydrogen sources, its ready availability, low toxicity, and reduced risk of explosions or other severe accidents make it a particularly attractive option for hydrogen storage. Furthermore, FA should be considered within a broader context of CO_2_ capture and reutilization, creating a potentially carbon-neutral cycle.^5^ This research direction holds great potential, particularly for the transformation of captured CO_2_ into value-added chemicals or for the controlled release of hydrogen through efficient homogeneous catalysis.^5^

Before the use of LOHCs, FA was already employed as a hydrogen source or reductor in decarboxylation reactions.^6–8^ Although its production still relies on fossil sources, such as the partial oxidation of naphtha and the carbonylation of methanol with hydrolysis of methyl formate,^9^ numerous studies demonstrate that renewable sources, such as biomass and hydrogenation of CO_2_ have great potential for FA production.^10–14^ This latter approach is particularly significant, as it represents a pathway for CO_2_ utilization, contributing to carbon neutrality.

FA dehydrogenation (FADH) requires the use of catalysts to lower the activation energy for this kind of reaction.^15,16^ In 2008, Beller and co-workers reported a selective FADH, catalyzed by commercially available Ru precursors with triphenylphosphine ligands in a FA/Et_3_N solution. The obtained gas was H_2_ and CO_2_, with no CO contamination as byproduct.^17^ Afterward, several catalysts based on complexes from late transition metals were evaluated for H_2_ production from FA, with Ru and Ir catalysts being the most extensively studied. Although Ru complexes generally exhibit lower activity in FADH compared with their Ir counterparts, they still demonstrate high efficiency, achieving turnover frequency (TOF) values of up to 10^6^ h^–1^ in optimized systems. This notable activity, combined with the lower cost of Ru relative to Ir, highlights Ru complexes as a promising alternative for practical applications.

During the FADH for H_2_ production, an equimolar amount of CO_2_ is generated. The capture, storage, and/or conversion of this CO_2_ into valuable chemical compounds can contribute significantly to carbon neutrality.^18,19^ One of the biggest challenges in CO_2_ transformation is its inherent chemical inertness, which requires highly efficient catalysts featuring high-energy orbitals capable of facilitating interaction with the electron-deficient carbon atom in CO_2_.^20^ Moreover, the reversibility of this process has been investigated as a potential solution for the sustainable production and storage of H_2_.^21^

Motivated by recent and promising results in FADH reactions as a hydrogen source, the present study aims to develop new and innovative catalytic systems based on monometallic cationic Ru complexes of the type [Ru­(κ^2^-OAc)­(dppb)­(N,N)], where N,N represents phenanthroline derivatives, as well as homobimetallic complexes of the type [(dppb)­(κ^2^-OAc)­Ru­(μ-L-C,N)­Ru­(κ^2^-OAc)­(dppb)]­OAc (Scheme 1). We investigate their catalytic performance in FADH, providing new mechanistic insights into the dehydrogenation pathway. Additionally, efforts for the storage of hydrogen as FA were performed from CO_2_ using 2-propanol as a hydrogen source.

**Scheme 1:**
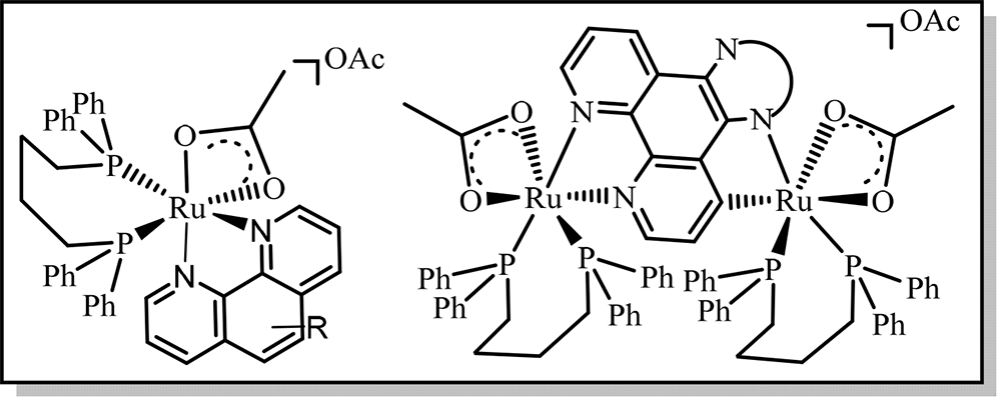
Scope of Catalyst Applied in FADH and TH of CO_2_

## 2 Results and Discussion

### 2.1 Synthesis and Characterization of the Complexes

The cationic complexes **1**–**5** were obtained from the reaction of [Ru­(OAc)_2_(dppb)] with 1.1 equiv of the respective ligand (HLa to HLe) in methanol at 60 °C (Scheme 2), according to the literature procedures.^22^ The homobimetallic complex **7** was synthesized from the derivative **5** with 1 equiv of [Ru­(OAc)_2_(dppb)] in toluene at 110 °C, in agreement with the procedure for **6**.^22^ All complexes were characterized by NMR, FTIR, UV–vis, and cyclic voltammetry measurements. Attempts to prepare the homobimetallic compounds from **3** and **4** failed. Thus, the presence of the phenyl and the phenanthroline moieties in **3** resulted in a mixture of two cyclometalated products, whereas in the case of **4**, the bulky *tert*-butyl group prevents the coordination of [Ru­(OAc)_2_(dppb)] to the imidazole nitrogen.

**Scheme 2:**
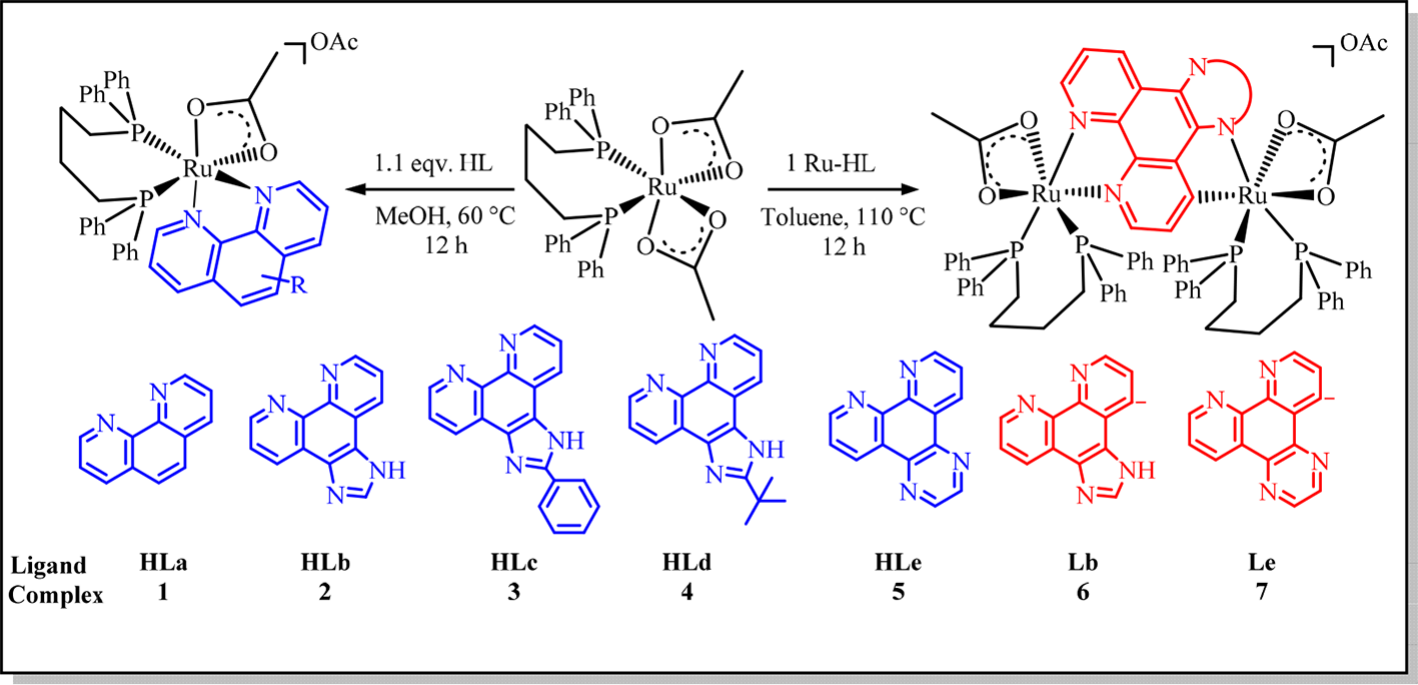
Synthesis of the Mono and Bimetallic Cationic Ru Complexes

The ^1^H NMR spectra of these complexes reveal nonequivalent *o*-phenanthroline hydrogens in the range δ 9.0–9.5 ppm (Figures S1–S4). A signal at δ 1.9 ppm for the noncoordinated acetate has been observed for all complexes, in agreement with the proposed structures of monocationic complexes, while the coordinated acetate appears as a singlet in the range δ ∼ 1.2 ppm. In addition, the homobimetallic **7** exhibits two signals for the coordinated acetate at δ 1.3 and δ 1.2 ppm. ^31^P­{^1^H} NMR of monometallic Ru complexes in CD_3_OD shows two doublets around δ 47.0 to 48.5 ppm, with the P atoms trans to the N and O atoms (Figures S5–S8). The ^31^P­{^1^H} NMR of **7** in CD_3_OD exhibits four doublets from δ 49.5 to 51.7 ppm, which differ from those of **6** (δ 60.0 to 50.0 ppm).^22^ ^13^C­{^1^H}­DEPTQ NMR measurements were recorded in CD_3_OD for the complexes (Figures S9–S12). The presence of a double of doublets at δ 213.0 ppm (dd, ^2^ *J*_CP_ = 17.7 Hz, ^2^ *J*_CP_ = 9.7 Hz; C–Ru) for **7** confirms the cyclometalated bimetallic complex.^23,24^

HRMS analysis of complexes **3**, **4**, **5**, and **7** in positive-ion mode revealed the corresponding molecular ions (Figures S13–S16). Additionally, complex **7** displayed an isotopic pattern consistent with a dinuclear species containing two Ru centers. The loss of one acetate ligand generated a dicationic complex (2+), which was also detected by HRMS.

FTIR spectra of the complexes (Figures S17–S19) were compared to their correspondent ligands and show the stretches corresponding to the coordinated ν­(C=O) in the region of 1600–1500 cm^–1^ overlapped with ν­(C=N) stretching. The phosphines are identified by the presence of the ν­(P–C) stretch at 1090 cm^–1^ in both complexes, followed by the ν­(Ru–O) stretch at 807 cm^–1^ for the coordinated acetate. UV–vis absorption spectra of the complexes in CH_2_Cl_2_ exhibit intraligand transitions in the UV region from the ligand-based π–π* transition (Figures S20–S23).^22,25^ Additionally, the complexes show moderate to intense bands in the visible region assigned to charge transfer from Ru­(II) dπ → NN pπ*and Ru­(II) dπ → dppb pπ*.^22,25^

All monometallic complexes exhibit a similar *E*_1/2_ value of ∼1.40 V for the Ru^II/III^ redox couple, with the **5** derivative showing the highest value (1.47 V) (Table 1). This increased potential can be attributed to the extended conjugation on HLe, which more efficiently withdraws electron density from the Ru center (Figures S24–S27). The homobimetallic complexes display two different processes: the lower potential value is related to the cyclometalated fragment, whereas the higher potential is attributed to the Ru–N,N fragment for both complexes. The higher potential for **6** (*E*_1/2_ = 1.40 V) is quite close to its monometallic fragment **2** (1.37 V), showing almost no influence on the electronic properties of the Ru–N,N fragment by the insertion of another cyclometalated Ru fragment. Interestingly, complex **7** exhibited a shift in *E*_1/2_ for its N,N fragment from 1.47 to 1.06 V, which is close to the potential of the Ru^II/III^ redox couple for the cyclometalated moiety (0.76 V).

**Table 1:** Cyclic Voltammetry Results for the Ru Complexes **1**–**7** ^a^ ^c^

complex	*E*_ap_	*E*_cp_(V)	*E*_1/2_(V)^b^	Δ*E*_p_(V)^d^
1	1.44	1.36	1.40	0.12
2	1.44	1.30	1.37	0.14
3	1.44	1.39	1.42	0.05
4	1.39	1.32	1.35	0.07
5	1.53	1.41	1.47	0.12
6	0.86^e^; 1.50^f^	0.75^d^; 1.30^f^	0.80^e^; 1.40^f^	0.06^e^; 0.20^f^
7	0.81^e^; 1.12^f^	0.72^e^; 1.00^f^	0.76^e^; 1.06^f^	0.09^e^; 0.12^f^

a Conditions: CH_2_Cl_2_, [Ru] = 1.0 × 10^–3^ mol L^–1^, *n*-Bu_4_NPF_6_ (0.1 mol L^–1^, supporting electrolyte), Ag/AgCl in saturated KCl (reference electrode).

b *E*_1/2_ = (*E*_ap_ + *E*_cp_)/2.

c Values obtained from ref 22.

d Δ*E*_p_ = *E*_ap_ – *E*_cp_.

e Cyclometalated fragment.

f N,N fragment.

### 2.2 Formic Acid Dehydrogenation

The monocationic phenanthroline Ru complexes were evaluated for the catalytic FADH reactions using a FA/catalyst molar ratio of 1000 (2.86 mmol/2.86 μmol). All complexes exhibited good solubility in FA, except for complexes **4** and **7**, which displayed moderate solubility. The gas products of the FA decomposition were flowed through a saturated NaOH solution to trap CO_2_, allowing for the collection of pure H_2_ (Scheme 3). Gas release was tracked over time, and the performance of the catalysts was evaluated by varying the amount of Et_3_N. TOF_20_ and TOF_50_ values were calculated at 20 and 50% yield, respectively, and the gas produced was analyzed by GC measurements to confirm its composition and purity (Figure S28).

**Scheme 3:**
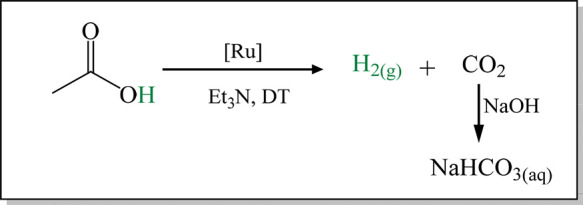
FADH Reactions Using Ru Complexes to Obtain Pure H_2(g)_

Initially, catalytic tests were performed in the absence of base, where the catalysts show minimal activity at 90 °C. Therefore, the effect of different amounts of Et_3_N was evaluated, and the data are shown in Table 2. The monometallic Ru complexes display low activity with low amounts of Et_3_N, and it was observed that H_2_ production increases with increasing Et_3_N/FA ratio, reaching maximum gas production at 75% of Et_3_N for **1** (entries 1–4).

**Table 2:** FADH Reactions Using the Monometallic Ru Complexes **1–7** ^a^

entry	cat	Et_3_N/FA (%)	yield (%)	time (min)	^b^TOF_20_	^c^TOF_50_	^d^TOF_20_/2
1	**1**	25	---	---	---		
2	**1**	50	7	1.2	---	---	
3	**1**	75	21	2.0	6428		
4	**1**	100	---	---	---		
5	**2**	75	29	3.3	5565		
6	**3**	75	7	3.6	---		
7	**4**	75	43	13	4285		
8	**5**	75	64	9	5741		
9	**6**	50	71	80	560	634	280
10	**6**	75	93	53	6036	2683	3018
11	**6**	100	86	96	4285	580	2142
12	**7**	50	71	80	886	671	443
13	**7**	75	93	74	4479	1363	2239
14	**7**	100	93	80	8130	2586	4065

a Reactions carried out using a molar ratio of FA/C = 1000 and different amounts of Et_3_N (in mol % relative to the substrate) at 90 °C.

b TOF_20_ (mol of H_2_/mol of cat./time­(*h*)) calculated at 20% of conversion.

c TOF_50_ calculated at 50% of yield.

d TOF_20_ calculated for each Ru center dividing TOF_20_/2.

The use of higher concentrations of amine is limited by the miscibility of the two components.^26^ Attempts to conduct the reaction using 25, 50, or 100 mol % of Et_3_N with complexes **2** to **5** result in negligible activity. Although all complexes exhibit high TOF values under optimal conditions, the overall H_2_ yield remains relatively low for most monometallic complexes. Complex **1** affords 21% yield (entry 3) with a TOF_20_ of 6428 h^–1^. Similarly, complex **2** gives 29% H_2_ with a TOF_20_ value of 5565 h^–1^ (entry 5) under the same conditions. These data show no considerable improvement in catalytic activity with the phenanthroline ligands containing the imidazolium moiety. The presence of a phenyl functional group at the end of the phenanthroline ligand in complex **3**, results in poor activity in the FADH reactions (entry 6), possibly due to the poor solubility of this complex in the reaction medium. On the other hand, the *tert*-butyl derivative **4** shows higher catalytic activity than **2** (entries 5 and 7), achieving around 43% yield in 13 min, with a TOF_20_ of 4285 h^–1^ when 75 mol % of Et_3_N was used. Finally, complex **5** exhibits the highest yield of H_2_ among the monometallic Ru catalysts, reaching 64% of yield in 9 min with a TOF_20_ of 5741 h^–1^ (entry 8).

Bimetallic complexes **6** and **7** were evaluated under the same conditions as the monometallic complexes but exhibited significantly different behavior. The variation in the Et_3_N concentration was systematically evaluated to determine the optimal Et_3_N/FA ratio for these catalysts. Both complexes **6** and **7** exhibit optimum performance when 75 mol % of Et_3_N is employed (entries 9 to 14), reaching 93% yield in both cases. Although using 100 mol % of Et_3_N results in a higher TOF compared to 75% for **7** (entries 13 and 14), the latter achieves the same yield (93%) in a shorter time. This occurs due to the decrease in the miscibility of FA in Et_3_N as the amount of base increases. Interestingly, the TOF_50_ for reactions involving complex **6** (2683 h^–1^) is nearly double that of **7** (1363 h^–1^) using 75 mol % of Et_3_N. This observation may be related to the lower solubility of **7** in the catalytic system. Table S2 summarizes the turnover number (TON) for the FADH reactions under optimized conditions. It is worth noting that the FADH carried out with the precursor [Ru­(OAc)_2_dppb] leads to poor conversion (<5%) under the optimized catalytic conditions.

In comparison with other systems, Ru-arene complexes containing different ancillary groups exhibited TOF values of the same order of magnitude, whereas Ru–P,N,P complexes achieved TOF values exceeding 200,000 h^–1^ under optimized conditions.^16,27–30^ Phosphorus-based ligands, in fact, play a crucial role in the activation of Ru–H species. Thus, the combination of tridentate chelating ligands with hydride-activating groups can enhance the catalytic performance in FA dehydrogenation. In our system, phosphine groups are fundamental for catalyst activation, while *N*,*N*-type ligands contribute to the stabilization of the active species during catalysis.

Figure 1 shows the kinetics of H_2_ volume produced over time for all complexes evaluated in the FADH under the optimal condition (75 mol % Et_3_N). The curves reveal similar profiles in initial times for all complexes, except for **3**, reaching 15 mL of H_2_ after ∼2 min. However, as the reaction progresses, the conversion rate to H_2_ decreases when monometallic complexes are employed, with **5** standing out for remaining active longer and producing higher H_2_ volumes. Both **6** and **7** derivatives show similar H_2_ production kinetics profiles: a fast initial rate for 10 min, followed by a slowdown and eventual stabilization. This behavior suggests that in the bimetallic species, both metal centers are initially active, but as the reaction progresses, the activity of the Ru–*N*,*N* fragment diminishes, while the Ru-cyclometalated center continues to catalyze the reaction. This hypothesis is supported by the observation that in monometallic catalysis, activity persists only briefly before deactivation, as evidenced by the kinetic curves in Figure 1.

**Figure 1:**
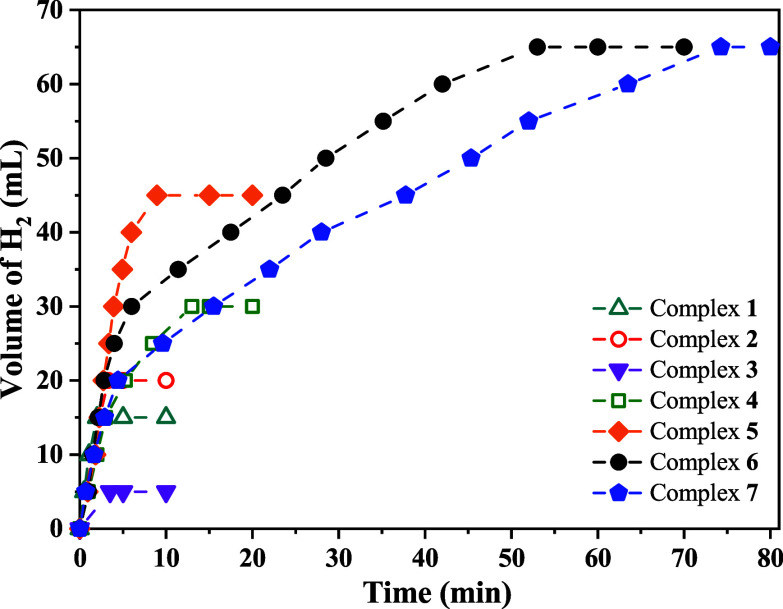
Volume of H_2_ over the time for Ru complexes using 75 mol % of Et_3_N at 90 °C; FA/Cat = 1000.

As complex **7** has a lower solubility than **6**, experiments with different solvents (0.5 mL) were conducted to understand if the difference in catalytic activity was due to solubility issues and to address the miscibility challenges between FA and Et_3_N (Table 3). For complex **6** (entries 1–4), TOF values decrease with the addition of solvents, but yield remains the same as in reactions without solvents (93%). By contrast, for complex **7**, the use of solvent improves both solubility and miscibility but results in slightly lower yields (entries 5–8).

**Table 3:** Solvent Effect on FADH Using Bimetallic Ru Complexes **6**–**7** ^a^

entry	complex	solvent	yield (%)	time (min)	TOF_20_ ^b^ (h^–1^)	TOF_50_ ^c^ (h^–1^)
1	**6**	---	93	53	6036	2683
2	**6**	DMF	93	60	3011	1593
3	**6**	dioxane	93	105	3456	1463
4	**6**	toluene	93	60	2727	1986
5	**7**	---	93	74	4479	1363
6	**7**	DMF	86	85	3591	983
7	**7**	dioxane	71	52	4761	3000
8	**7**	toluene	86	102	4285	1913

a Reactions conducted in different solvents (0.5 mL) using FA/Cat = 1000 and Et_3_N/FA = 75 mol % at 90 °C.

b TOF_20_ calculated at 20% of yield.

c TOF_50_ calculated at 50% of yield.

Complex **6** exhibits no catalytic activity at temperatures below 90 °C. The formation of an azeotropic mixture in the toluene-Et_3_N-FA system enabled an increase in the reaction temperature within the range of 90 to 110 °C.^31,32^ Thus, toluene was subsequently used in FADH reactions at various temperatures to calculate the thermodynamic parameters (Table S1). The kinetic curves of the H_2_ volume produced were monitored over time (Figure 2), and the corresponding data are summarized in Table S1. The rate of reaction shows a clear increase from 90 to 110 °C, with the TOF_20_ value approximately two times higher, while the time required for complete conversion decreases by nearly 3-fold. Furthermore, a 100% yield of H_2_ is obtained at temperatures above 90 °C, demonstrating the positive effect of the temperature on both reaction rate and overall conversion. In the 90–110 °C range, pseudo-first-order rate constants were obtained from linear fits of −ln­(1 – X) versus the activation-corrected time *t*′ = *t* – *t*_act_ *t*′ intercept constrained to zero, yielding *k*_obs_ after brief activation periods. Arrhenius analysis of ln *k* versus 1/*T* (*k*–1) gave *E*_a_ = 60.3 kJ mol^–1^, in accordance with other Ru complexes for FADH.^33–36^ Eyring analysis of ln (*k*/*T*) versus 1/*T* (with *k* in s^–1^) afforded Δ*H* ^⧧^ = 57.2 kJ mol^–1^ and Δ*S* ^⧧^ = −149.6 J mol^–1^ K^–1^ (−35.8 e.u.), consistent with *E*_a_ ≈ Δ*H* ^⧧^ + RT at ∼373 K. The resulting free energies of activation span 111–115 kJ mol^–1^ across 363–383 K (e.g., Δ*G* ^⧧^ = 113.0 kJ mol^–1^, 27.0 kcal mol^–1^, at 373.15 K), indicating a moderately slow, enthalpically modest yet entropically disfavored rate-limiting step. The large negative Δ*S* ^⧧^ points to an ordered transition state and/or preassociation of reaction partners, consistent with a coordination-controlled pathway preceding H_2_ evolution.

**Figure 2:**
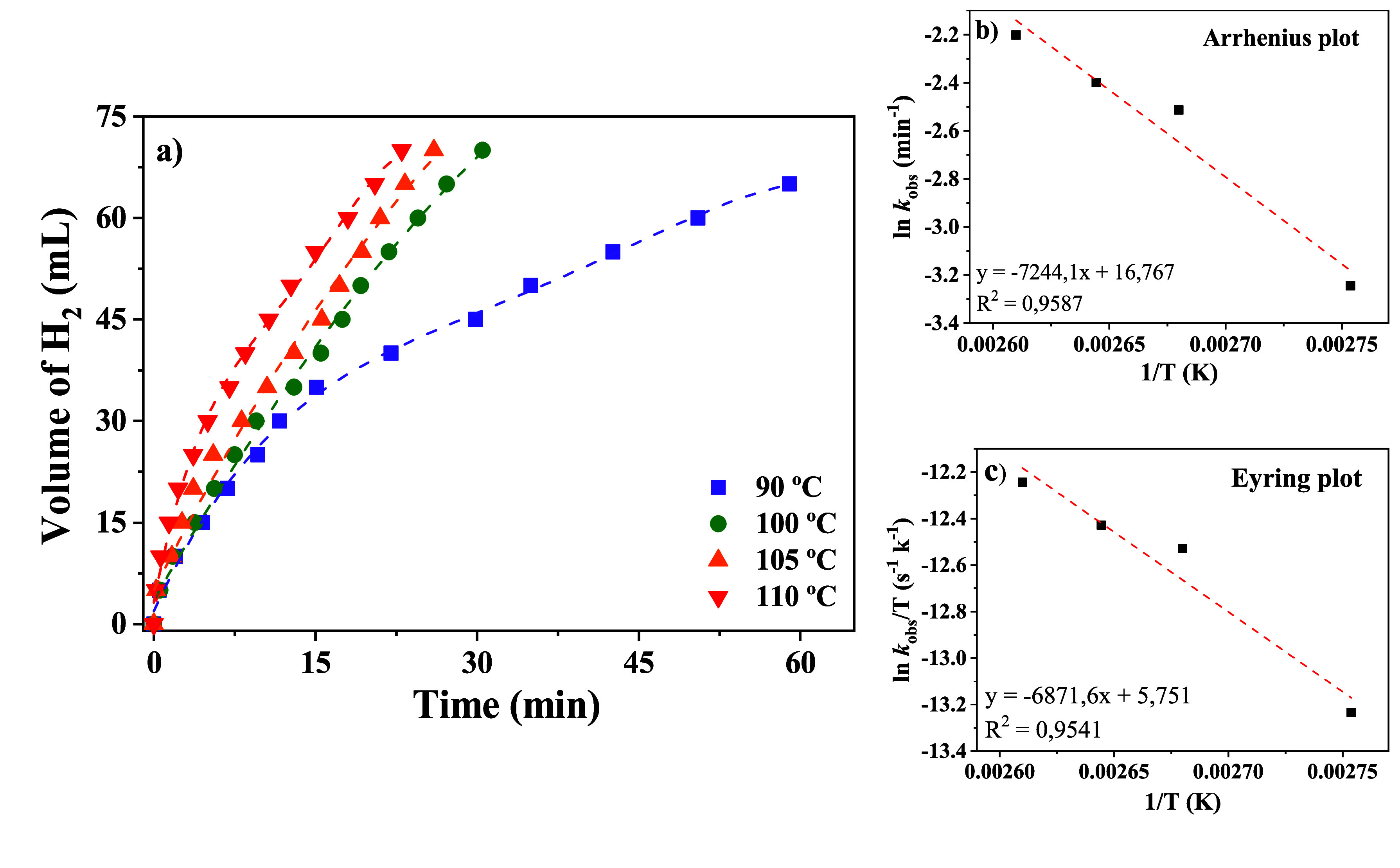
(a) FADH using **6** in toluene at different temperatures. (b) *E*_a_ determination using Arrhenius plot. (c) Δ*S* ^⧧^ and Δ*H* ^⧧^ determination using Eyring plot.

The stability of the catalytic system for FADH reactions using **6** and Et_3_N was demonstrated in toluene at 110 °C through a 10 day recycling process, aiming to assess its potential for large-scale applications. A linear increase in accumulated TON values was observed from the results obtained in each catalytic cycle, as all reactions provided a 100% yield (Figure 3). Additionally, the TOF_50_ shows no significant oscillations, with values ranging from 3650 to 4285 h^–1^, demonstrating that the catalytic system remains stable under catalytic conditions, even at elevated temperatures.

**Figure 3:**
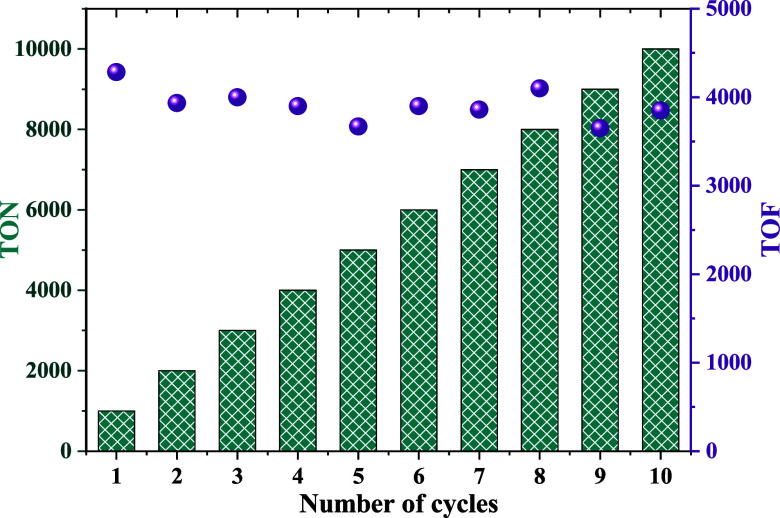
Recycling process of the catalytic system using **6** and 75 mol % of Et_3_N in toluene (0.5 mL) at 110 °C; FA/cat = 1000. TON values are represented by green columns, while TOF values are represented by purple balls.

To elucidate the processes involved in the FADH reactions of [Ru­(OAc)­(dppb)­(*N*,*N*)] derivatives, NMR studies were performed using complex **2** and varying amounts of FA in CD_3_OD, since this complex represents the *N*,*N* fragment of binuclear complex **7**, which exhibited the best catalytic performance in FADH and was employed for the determination of thermodynamic parameters. Initially, a control experiment involving the reaction between **2** and different amounts of FA was monitorated by ^31^P­{^1^H} NMR at 25 °C (Figure 4). Complex **2** exhibits low solubility in CD_3_OD; however, the addition of 1 equiv of FA completely solubilizes the complex, resulting in 100% conversion to a new species. Further addition of 20 equiv of FA did not produce any additional product. These results, consistent with the ^1^H NMR (Figures S29 and S30), reveal the formation of a new species, associated with the substitution of OAc^–^, both as a coordinated ligand and as a counterion, by formate (2d, δ_P_ = 47.76 ppm, ^2^ *J*_P,P_ = 33.73 Hz). Heating the solution for 10 min at 90 °C led to the formation of the Ru-monohydride species [RuH­(κ^1^-CHO_2_)­(dppb)­(HLb)], with the hydride in a *fac* arrangement with respect to the two P atoms, as confirmed by the double of doublets at δ_H_ **–**12.53 ppm (dd, ^2^ *J*_H,P_ = 20.78 Hz, ^2^ *J*_H,P_ = 25.84 Hz) (Figure S30).^22,37^ Further heating for 3 h led to the formation of a second monohydride species, in which the hydride is arranged meridionally with respect to the two phosphorus atoms, as evidenced by the signal at δ_H_ −6.04 ppm (^2^ *J*_H,P_ = 23.17 Hz and ^2^ *J*_H,P_ = 99.38 Hz) (Figure S30),^21,30^ showing coupling constants typical of a *cis* and *trans* H–Ru–P arrangement.^38^ Prolonged exposure of the solution at 90 °C led to a higher conversion to the compound with hydride *trans* to the P atom. ^1^H–^31^P HMBC NMR measurements confirmed the correlation between the proton signals at δ_H_ −12.53 with the signals at δ_P_ 56.9 and 25.5 ppm for H–Ru–P *cis*, whereas the resonance at δ_H_ −6.04 ppm is coupled with the signals at δ_P_ 57.5 and 10.0 ppm for H–Ru–P *trans* (Figure S31). Attempts to perform 2D ^1^H–^1^H correlation spectroscopy (COSY) experiments did not reveal any cross-peaks between the hydride signals, thus ruling out the possibility of a Ru-dihydride species. These assignments reveal two distinct species with two distinct geometry.

**Figure 4:**
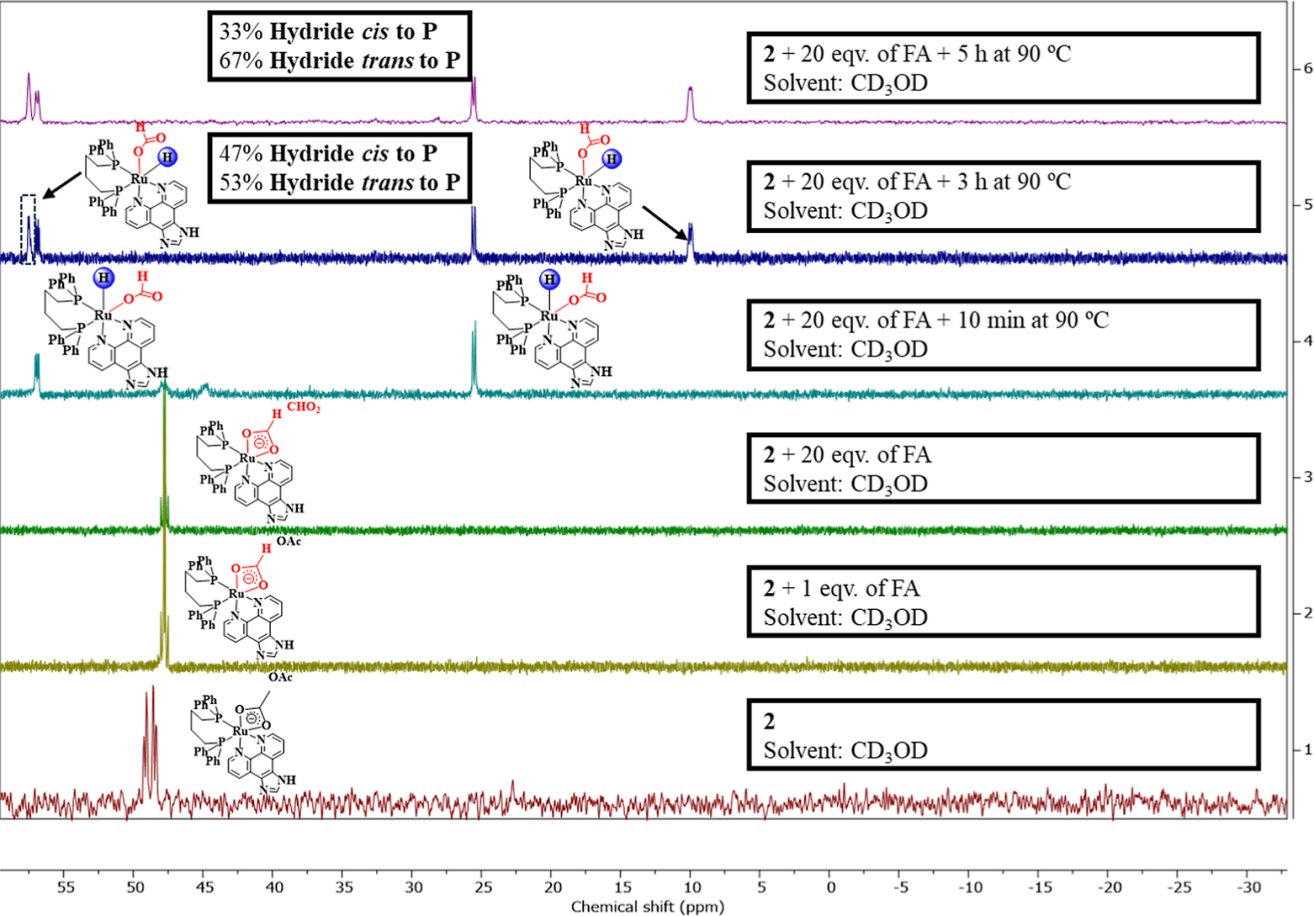
^31^P­{^1^H} NMR control experiments of the formation of Ru-hydride species in CD_3_OD from complex **2** and FA at 90 °C—(δ in ppm).

Complementary data were obtained from density functional theory (DFT) calculations to identify the intermediates involved in the FADH mechanism (Scheme 4). The formation of intermediates **2** ^**II**^ and **2** ^**III**^ derivatives is associated with a gain in Gibbs free energy, which is then followed by a decrease upon coordination of a formate ligand (**2** ^**IV**^ derivatives). The approach of a proton to generate the Ru–H_2_ species (**2** ^**V**^ derivatives) results in a drastic increase in the system’s energy. In all cases, the Gibbs free energy is higher for the Ru–H species bearing the hydride *trans* to the phosphorus atom.

**Scheme 4:**
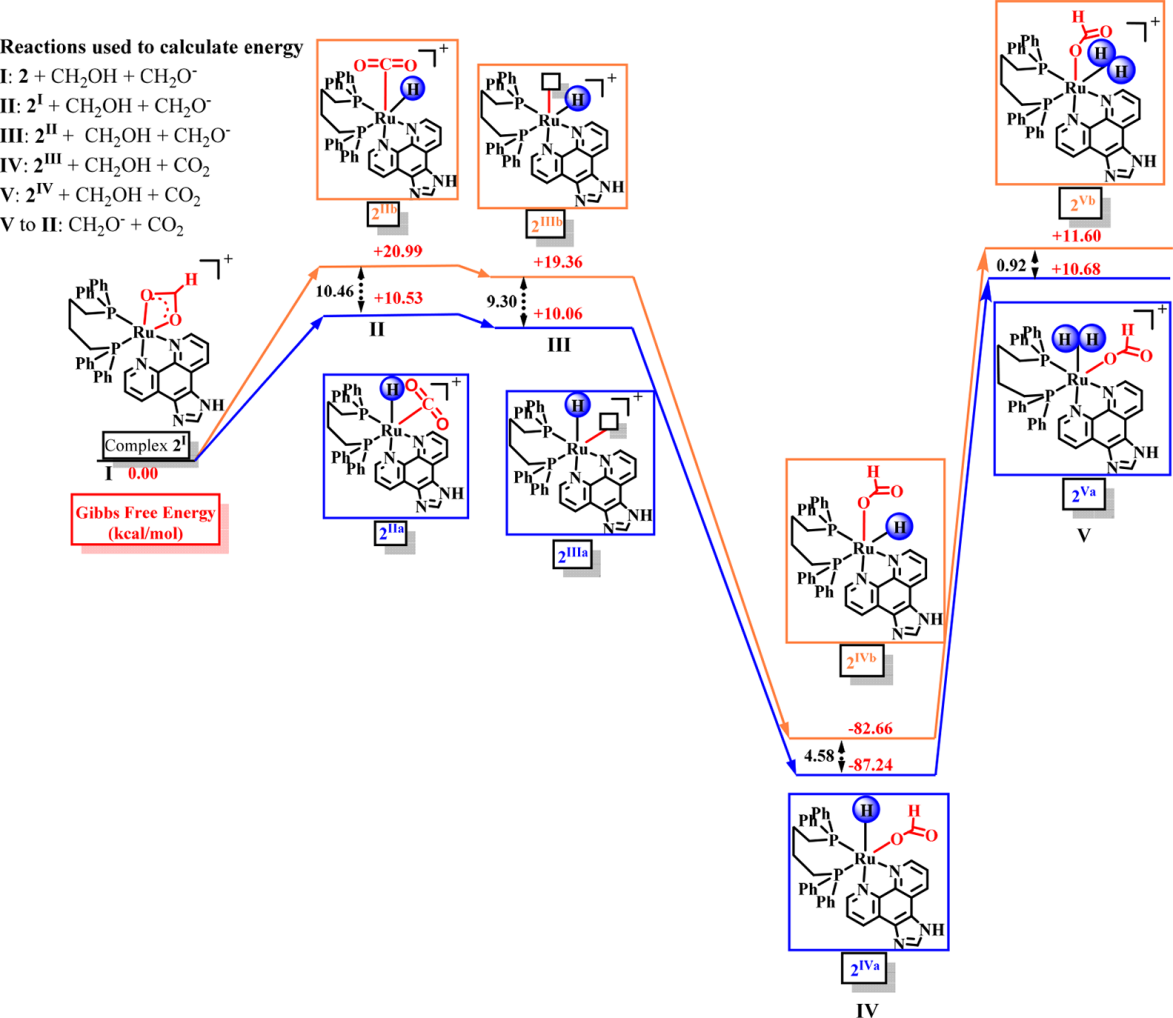
Gibbs Free Energy of the Intermediate Species in the FADH Mechanism

Based on these findings, Figure 5 illustrates the proposed two concomitant mechanisms on FADH using complex **2**, which are further supported by DFT calculations. Initially, the formate from the mixture of FA/Et_3_N reacts with Ru complex, affording the Ru-formate derivative (**2** ^**I**^) by acetate displacement in which heating the system leads to the formation of a Ru-monohydride species (**2** ^**IIa**^ and **2** ^**IIb**^), being the hydride *cis*-positioned to the P atoms, the kinetic and most stable product. The labilization of the CO_2_ molecule from the Ru center affords the most energetic species (**2** ^**IIIa**^ and **2** ^**IIIb**^), which are readily converted into **2** ^**IV**^ derivatives by the coordination of a formate. The interaction of **2** ^**IV**^ derivatives with an H^+^ in the medium affords the intermediate Ru–H_2_ species (**2** ^**V**^), releasing H_2(g)_ to regenerate the catalytic cycles, affording an equimolar amount of CO_2_ as a product.

**Figure 5:**
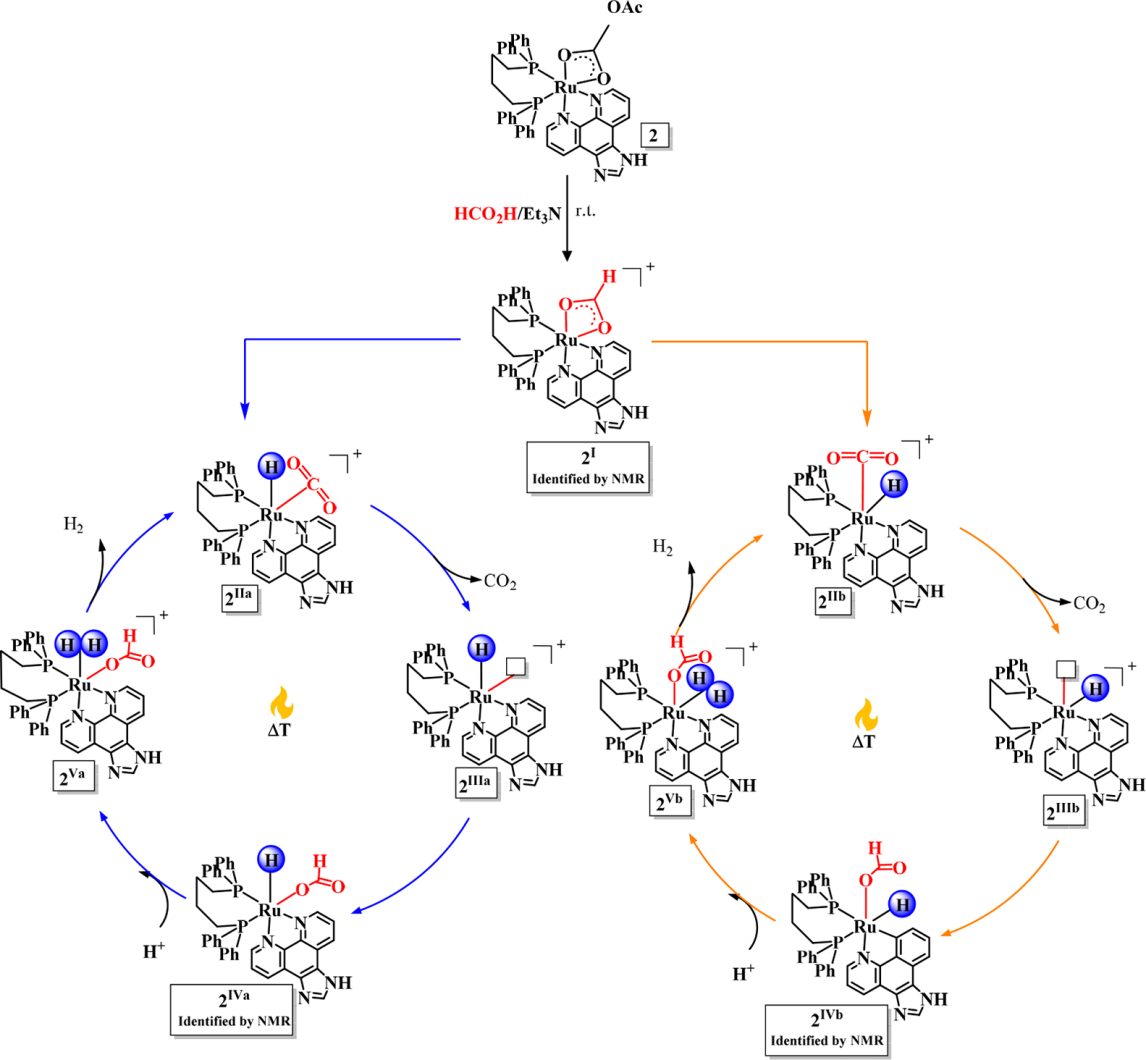
Mechanism proposal for FADH using cationic [Ru­(OAc)­(dppb)­(N,N)] derivatives.

Attempts to investigate the mechanism involving FADH in dinuclear complexes were also carried out for complex **6**, using a similar approach to that employed for **2**. However, multiple signals corresponding to Ru–H species were identified in both the ^1^H and ^31^P­{^1^H} NMR spectra (Figure S32), on account of the two Ru centers, which are involved in the substitution of the acetate with the formate ligand and β-hydride elimination reactions. Furthermore, the thermodynamic parameters showed that the Δ*S* ^⧧^ value for complex **6** (−149.6 J mol^–1^ K^–1^) is approximately 1.7× higher than that of complex **2** (−90.9 J mol^–1^ K^–1^) (Figure S33 and Table S3), indicating nearly twice the degree of organization for the dinuclear species, which is consistent with a system containing two active sites. Nonetheless, based on the studies on monometallic complex **2**, it is reasonable that the mechanism proceeds through analogous steps, with a cooperative effect between the two metal centers that enhances the overall catalytic efficiency.

### 2.3 Transfer Hydrogenation Reactions of CO_2_

The monocationic ruthenium complexes were also evaluated in transfer hydrogenation (TH) of CO_2_, using *i*PrOH as a hydrogen donor, which represents a complementary process to FADH and offers a potential strategy for achieving carbon neutrality in hydrogen production and storage cycles. Control experiments conducted in the absence of any catalyst under 50 bar of CO_2_ at 100 °C in a 0.5 M KOH solution using a 20:5 mL H_2_O/*i*PrOH mixture failed to produce FA (Table 4, entry 1). As expected, only potassium bicarbonate (KHCO_3_) was formed as a result of the direct reaction between CO_2_ and KOH (Figure S34). The performance of all synthesized complexes was evaluated under the same conditions (entries 2–8), and the reaction products were analyzed by ^1^H NMR using D_2_O as a solvent and isonicotinic acid as an internal standard (Figure S35).

**Table 4:**
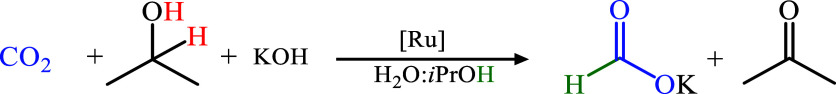
Transfer Hydrogenation of CO_2_ Using Ru Complexes **1–7** ^a^

entry	complex	*n*_HCOOK_/10^–6^	TON
1	---	---	---
2	**1**	17	1.3
3	**2**	22	1.7
4	**3**	27	2.1
5	**4**	20	1.5
6	**5**	27	2.1
7	**6**	31	2.4
8	**7**	24	1.8

a Reactions conducted using 13 μmol of catalyst, 50 bar of CO_2_, 0.5 M KOH in H_2_O/*i*PrOH (20:5 mL) at 100 °C for 22 h.

b TON = mol of formate/mol of catalyst.

The complexes show low activity in the TH of CO_2_, with TON values ranging between 1.3 and 2.4. Interestingly, neither the modifications to the Phen ligand nor the incorporation of a second Ru fragment significantly affected the catalytic performance in this reaction. These results are consistent with other similar complexes based on bipyridine ligands previously reported in the literature.^39,40^ Ohnishi et al. have investigated CO_2_ hydrogenation catalyzed by Ru complexes by DFT calculations, showing that the rate-determining step in the reaction is related to the insertion of CO_2_ into the Ru–H bond. Our previous studies have shown that monocationic Ru complexes based on dppb, OAc, and phenanthroline derivatives are capable of forming Ru–H species using *i*PrOH/NaO*i*Pr as the hydrogen source.^22^ This suggests that the rate-limiting step is not related to the formation of the Ru–H species but rather to the activation of CO_2_.

Recent computational studies suggest that ligands with strong σ-donor character and cooperative functionality (such as PNP or CNN pincer ligands) are more effective in promoting CO_2_ insertion into the Ru–H bond.^41,42^ Furthermore, the reaction conditions employed (H_2_O/*i*PrOH as the solvent) may not be optimal for CO_2_ solubilization, limiting its availability in the coordination sphere of the catalyst. The structural modifications had a minimal impact on the performance in TH of CO_2_, with all complexes showing similarly low activity. This suggests that the rate-limiting step in this reaction (likely the insertion of CO_2_ into the Ru–H bond) is not significantly influenced by the electronic or structural variations introduced in these complexes.

## 3 Conclusions

In this study, we have successfully synthesized and characterized a series of new monocationic ruthenium complexes containing phenanthroline derivatives and their corresponding dinuclear analogs. All complexes were fully characterized by spectroscopic and electrochemical techniques, which confirmed their proposed structures. The catalytic evaluation of these complexes in FADH revealed that the dinuclear systems, particularly **6** and **7**, exhibit superior catalytic performance compared to their monometallic counterparts, achieving up to 100% conversion under optimized conditions. The complex **6** demonstrated excellent stability, maintaining consistent TOF values through multiple catalytic cycles, which highlights its potential for practical applications in hydrogen production from FA. Mechanistic investigations using **2** provided valuable insights into the FADH pathway, revealing the formation of two key monohydride species through the substitution of acetate by formate followed by β-elimination. These findings contribute to our understanding of the fundamental processes involved in FADH catalyzed by ruthenium complexes. While the complexes showed promising activity in FADH, their performance in the complementary TH of CO_2_ with 2-propanol was limited, with TON values not exceeding 2.4. This disparity in catalytic activity between the forward (FADH) and reverse (TH of CO_2_) reactions highlights the challenges in developing efficient dual-function catalysts for reversible interconversion between CO_2_ and FA.

## 4 Experimental Part

### 4.1 General Remarks

Unless otherwise stated, all syntheses and manipulations were performed under an argon atmosphere following standard Schlenk techniques. All solvents were carefully dried by standard methods and distilled under argon prior to use. Reactants were purchased from Sigma-Aldrich and used without further purification. The ligands HLa to HLe,^43,44^ and the complexes **1**, **2**, and **6** ^22^ were obtained following previous protocols. Infrared spectra were obtained on a PerkinElmer Frontier instrument equipped with a diamond ATR module. The absorption spectra were recorded on a Shimadzu (model UV-1800) spectrophotometer, using 1 cm path length quartz cells. Electrochemical measurements were performed using an Autolab PGSTAT204 potentiostat with a stationary platinum disk and a wire as the working and auxiliary electrodes, respectively. The reference electrode was Ag/AgCl. The measurements were performed at 25 °C ± 0.1 in CH_2_Cl_2_ with 0.1 mol L^–1^ of *n*-Bu_4_NPF_6_. NMR measurements were recorded on an Avance III HD NMR 400 spectrometer. Chemical shifts are reported in ppm (δ). Elemental analyses were performed with a PerkinElmer CHN2400 instrument. Exact mass spectra were recorded on an Orbitrap Thermo QExactive using an electrospray ion source in positive mode. All calculations were carried out using the Gaussian16 suite of programs.^45^ The structures were optimized by the DFT method, using the PBE0 hybrid functional.^46^ This functional mixes the Perdew–Burke–Ernzerhof (PBE) and Hartree–Fock exchange energies, along with the full PBE correlation energy. The basis set used to build the molecular orbitals were LANL2DZ (Los Alamos National Laboratory 2 double-ζ) for ruthenium^47^ and def2-SV­(P)^48^ for the remaining atoms. The Hessian matrix was calculated for the optimized structures in order to verify the nature of the stationary state.

### 4.2 Synthesis of [Ru­(κ^2^-OAc)­(dppb)­(HLc)]­OAc (**3**)

The complex **3** was synthesized following the procedure described for **1**. [Ru­(OAc)_2_(dppb)] (0.400 g, 6.2 × 10^–4^ mol) and 1.1 equiv of 2-phenyl-1*H*-imidazo­[4,5-*f*]­[1,10]­phenanthroline (HLc) were added to a Schlenk flask. The system was purged with argon by applying five vacuum/argon cycles, followed by the addition of 10 mL of MeOH. The mixture was allowed to react for 12 h at 60 °C, then cooled to room temperature, and reduced to ∼1 mL, followed by the addition of 10 mL of diethyl ether. The precipitate was filtered and washed three times with diethyl ether (3 × 5 mL) to obtain a red microcrystalline powder (535 mg, 92% yield). UV–vis (CH_2_Cl_2_): λ_max_ nm (ε_max_ [10^3^ mol L^–1^ cm^–1^]): 334 (3.76 × 10^1^), 395 (9.47), 460 (4.86). FTIR (cm^–1^): 3111–3012 ν­(C–H)_aromatic_, 2981–2827 ν­(C–H), 1640–1500 ν­(overlapped C=O e C=N), 1461 ν­(C=O), 1090 ν­(P–C), 694 ν­(Ru–O). ^1^H NMR (400.1 MHz, CD_3_OD, 25 °C): δ = 9.02 (d, ^3^ *J*_HH_ = 4.89 Hz, 1H; ο-Phen), 8.92 (t, 2H, ^3^ *J*_HH_ = 8.23 Hz; Phen), 8.50 (d, 2H, ^3^ *J*_HH_ = 5.36 Hz; Phen), 8.28 (d, 2H, ^3^ *J*_HH_ = 7.01 Hz; Phen), 8.06–7.90 (m, 5H; aromatic protons), 7.72–7.55 (m, 7H; Phen), 7.53–7.46 (t, 2H; ^3^ *J*_HH_ = 7.56 Hz), 7.44–7.34 (m, 4H; aromatic protons), (t, 2H; ^3^ *J*_HH_ = 8.31 Hz), 6.42 (t, ^3^ *J*_HH_ = 7.57 Hz, 1H; Ph), 6.18 (t, ^3^ *J*_HH_ = 7.57 Hz, 2H; Ph), 5.69 (t, _3_ *J*_HH_ = 8.40 Hz, 2H; Ph), 3.26 (m, 1H; PCH_2_), 2.77 (m, 1H; PCH_2_), 2.45 (m, 3H; CH_2_), 2.10 (m, 2H; CH_2_), 1.91 (s, 3H; free CH_3_CO), 1.67 (m, 1H; CH_2_), 1.19 (s, 3H; CH_3_CO coordinated). ^13^C­{^1^H} NMR (100.6 MHz, CD_3_OD, 25 °C): δ = 178.0 (s; OCOCH_3_), 176.3 (s; OCOCH_3_), 158.0–122.0 (m; aromatic carbons), 36.4–20.0 (m, PCH_2_ and CH_3_CO). ^31^P­{^1^H} NMR (162 MHz, CD_3_OD, 25 °C): δ = 48.1 (d, ^2^ *J*_PP_ = 33.68 Hz), 47.5 (d, ^2^ *J*_PP_ = 32.93 Hz). HRMS (ESI+) calcd for [C_49_H_43_N_4_O_2_P_2_^102^Ru]^+^: 883.1899; found, 883.1905. Elemental analysis for C_51_H_46_N_4_O_4_P_2_Ru: Calcd: C, 65.03; H, 4.92; N, 5.95. Found: C, 65.31; H, 5.18; N, 6.17.

### 4.3 Synthesis of [Ru­(κ^2^-OAc)­(dppb)­(HLd)]­OAc Where HLd (**4**)

Complex **4** was prepared following the procedure described for **3** using 2-(*tert*-butyl)-1*H*-imidazo­[4,5-*f*]­[1,10]­phenanthroline (HLd) instead of HLc, obtaining the product as a red microcrystalline powder (526 mg, 90% yield). UV–Vis (CH_2_Cl_2_): λ_max_ nm (ε_max_ [10^3^ mol L^–1^ cm^–1^]): 288 (2.004 × 10^1^), 389 (1.66 × 10^1^), 350 (7.26), 435 (3.30). FTIR (cm^–1^): 3108–3031 ν­(C–H)_aromatic_, 3031–2851 ν­(C–H), 1628–1485 ν (overlapped signals C=O e C=N), 1454 ν­(C=O), 1090 ν­(P–C), 691 ν­(Ru–O). ^1^H NMR (400.1 MHz, CD_3_OD, 25 °C): δ = 9.0 (m, 1H; ο-Phen), 8.9 (t, 2H, ^3^ *J*_HH_ = 7.4 Hz; Phen), 8.5 (d, 2H, ^3^ *J*_HH_ = 5.3 Hz; Phen), 8.0–7.9 (m, 4H; aromatic protons), 7.9–7,8 (m, 1H; Phen), 7.7–7.5 (m, 4H; aromatic protons), 7.5–7.2 (m, 8H; aromatic prótons), 6.3 (t, ^3^ *J*_HH_ = 6.4 Hz, 1H; Ph), 6.1 (t, ^3^ *J*_HH_ = 6.8 Hz, 2H; Ph), 5.7 (t, ^3^ *J*_HH_ = 8.2 Hz, 2H; Ph), 3.2 (m, 1H; PCH_2_), 2.7 (m, 1H; PCH_2_), 2.4 (m, 3H; CH_2_), 2.0 (m, 2H; CH_2_), 1.9 (s, 3H; free CH_3_CO), 1.6 (overlapped m and s, 10H; CH_2_ and *tert*-butyl), 1.2 ppm (s, 3H; CH_3_CO). ^13^C­{^1^H} NMR (100.6 MHz, CD_3_OD, 25 °C): δ = 188.8 (s; OCOCH_3_), 178.8 (s; OCOCH_3_), 164.0–122.0 (m; aromatic carbons), 33.8 ppm (s; CH_2_), 28.49 (s; *tert*-butyl) 28.1 (d, ^1^ *J*_CP_ = 26.6 Hz; PCH_2_), 25.9 (d, ^1^ *J*_CP_ = 28.5 Hz; PCH_2_), 24.6 (s; CH_2_), 22.9 (s; CH_3_CO), 22.7 (s; CH_3_CO), 21.8 ppm (s; CH_2_). ^31^P­{^1^H} NMR (162 MHz, CD_3_OD, 25 °C): δ = 48.2 (d, ^2^ *J*_PP_ = 33.7 Hz), 47.5 ppm (d, ^2^ *J*_PP_ = 34.0 Hz). HRMS (ESI+) calcd for [C_47_H_47_N_4_O_2_P_2_^102^Ru]^+^: 863.2217; found, 863.2219. Elemental analysis for C_49_H_50_N_4_O_4_P_2_Ru: Calcd: C, 63.83; H, 5.47; N, 6.08. Found: C, 63.98; H, 5.66; N, 6.29.

### 4.4 Synthesis of [Ru­(κ^2^-OAc)­(dppb)­(HLe)]­OAc Where HLe (**5**)

Complex **5** was prepared following the procedure described for **4** using pyrazino­[2,3-*f*]­[1,10]­phenanthroline (HLe) instead of HLd, obtaining the product as a red microcrystalline powder (403 mg, 74% yield). UV–vis (CH_2_Cl_2_): λ_max_ nm (ε_max_ [10^3^ mol L^–1^ cm^–1^]): 256 (4.12 × 10^1^), 295 (1.93 × 10^1^), 340 (3.94), 415 (4.73), 490 (1.66). FTIR (cm^–1^): 3121–2997 ν­(C–H)_aromatic_, 2961–2828 ν­(C–H), 1609–1493 ν­(overlapped C=O e C=N), 1458 ν­(C=O), 1090 ν­(P–C), 693 ν­(Ru–O). ^1^H NMR (400.1 MHz, CD_3_OD, 25 °C): δ = 9.4 (d, ^3^ *J*_HH_ = 8.2 Hz, 1H; ο-Phen), 9.4 (d, ^3^ *J*_HH_ = 8.2 Hz, 1H; ο-Phen), 9.2 (m, 1H), 9.1 (dd, 2H), 8.6 (d, ^3^ *J*_HH_ = 5.4 Hz, 1H; Prz), 8.1–7.9 (m, 5H; aromatic protons), 7.7–7.6 (m, 5H; aromatic protons) 7.5–7.4 (m, 3H; Phen), 7.46–7.40 (m, 4H; aromatic protons), 7.3–7.2 (t, ^3^ *J*_HH_ = 8.6 Hz, 2H; Prz), 6.9 (t, ^3^ *J*_HH_ = 7.4 Hz, 1H; Ph), 6.1 (t, ^3^ *J*_HH_ = 7.1 Hz, 2H; Ph), 5.7 (t, ^3^ *J*_HH_ = 8.4 Hz, 2H; Ph), 3.2 (m, 1H; PCH_2_), 2.8 (m, 1H; PCH_2_), 2.5 (m, 3H; CH_2_), 2.1 (m, 2H; CH_2_), 1.9 (s, 3H; free CH_3_CO), 1.6 (m, 1H; CH_2_), 1.2 (s, 3H; coordinated CH_3_CO). ^13^C­{^1^H} NMR (100.6 MHz, CD_3_OD, 25 °C): δ = 190.0 (s; OCOCH_3_), 180.0 (s; OCOCH_3_), 160.0–125.0 (m; aromatic carbons), 29.5 (d, ^1^ *J*_CP_ = 27.3 Hz; PCH_2_), 27.3 (d, ^1^ *J*_CP_ = 29.6 Hz; PCH_2_), 26.0 (s; CH_2_), 24.2 (s; CH_3_CO), 24.1 (s; CH_3_CO), 23.2 ppm (s; CH_2_). ^31^P­{^1^H} NMR (162 MHz, CD_3_OD, 25 °C): δ = 47.8 (d, ^2^ *J*_PP_ = 34.0 Hz), 47.0 (d, ^2^ *J*_PP_ = 33.4 Hz). HRMS (ESI+) calcd for [C_44_H_39_N_4_O_2_P_2_^102^Ru]^+^: 819.1586; found, 819.1587. Elemental analysis for C_46_H_42_N_4_O_4_P_2_Ru: Calcd: C, 62.94; H, 4.82; N, 6.38. Found: C, 63.22; H, 4.93; N, 6.59.

### 4.5 Synthesis of Homobimetallic [(dppb)­(κ^2^-OAc)­(Ru­(HLe-μ-L5-NC)­Ru­(κ^2^-OAc)­(dppb)]­OAc (**7**)

Complex **7** was synthesized by following the procedure described for **6**. A 50 mL Schlenk flask containing complex **5** (202 mg, 2.3 × 10^–4^ mol) and [Ru­(OAc)_2_(dppb)] (0.148 mg, 2.3 × 10^–4^ mol, 1 equiv) was filled by argon by applying five vacuum/Ar cycles. Toluene (10 mL) previously degassed was added to the flask, and the mixture was allowed to react for 12 h under 110 °C. The system was cooled, and the solution was reduced to ∼1 mL. Diethyl ether (10 mL) was added to the flask, and the precipitate was filtered. The solid was washed with diethyl ether (3 × 5 mL) and dried under a vacuum. Red microcrystalline powder (337 mg, 83% yield). UV–vis (CH_2_Cl_2_): λ_max_ nm (ε_max_ [10^3^ mol L^–1^ cm^–1^]): 263 (3.39 × 10^1^), 297 (2.10 × 10^1^), 334 (7.05), 434 (3.43), FTIR (cm^–1^): 3117–2997 ν­(C–H)_ar_, 2988–2828 ν­(C–H), 1625–1486 ν­(overlapped C=O e C=N), 1433 ν­(C=O), 1095 ν­(P–C), 694 ν­(Ru–O). ^1^H NMR (400.1 MHz, CD_3_OD, 25 °C): δ = 9.1 (dd, ^3^ *J*_HH_ = 1.4 Hz, 1H; ο-Phen), 9.0 (m, 1H; ο-Phen), 8.9 (t, ^3^ *J*_HH_ = 2.6 Hz, 1H; C=N, Prz), 8.8 (m, 1H; C=N, Prz), 8.1 (t, ^3^ *J*_HH_ = 8.0 Hz, 2H; aromatic protons) 8.0–7.9 (m, 4H; aromatic protons), 7.7–7.6 (m, 8H; aromatic protons), 7.6–7.5 (m, 4H; aromatic protons), 7.4–7.3 (m, 9H; aromatic protons), 7.3–7.2 (m, 4H; aromatic protons) 6.9 (2d, ^3^ *J*_HH_ = 6.1 Hz, 2H; aromatic protons), 6.2 (m, 6H; aromatic protons), 5.7 (t, ^3^ *J*_HH_ = 7.4, 2H; Ph), 5.7 (t, ^3^ *J*_HH_ = 5.6 Hz, 2H; Ph), 3.1 (m, 1H; PCH_2_), 3.0 (m, 1H; PCH_2_), 2.7 (m, 1H; PCH_2_), 2,6–2.0 (m, 10H; PCH_2_), 1.9 (s, 3H; free CH_3_CO), 1.9–1.5 (m, 3H; PCH_2_), 1.3 (s, 3H; coordinated CH_3_CO), 1.2 ppm (s, 3H; coordinated CH_3_CO). ^13^C­{^1^H} NMR (100.6 MHz, CD_3_OD, 25 °C): δ = 213.0 (dd, ^2^ *J*_CP_ = 17.7 Hz, ^2^ *J*_CP_ = 9.7 Hz; C–Ru), 190.1 (s; OCOCH_3_), 187.8 (s; OCOCH_3_), 178.3 (s; OCOCH_3_), 153.0–125.0 (m; aromatic carbons), 30.7 (d, ^1^ *J*_CP_ = 26.8 Hz; PCH_2_), 30.0 (d, ^1^ *J*_CP_ = 26.8 Hz; PCH_2_), 28.0 (d, ^1^ *J*_CP_ = 28.2 Hz; PCH_2_), 27.0 (d, ^1^ *J*_CP_ = 32.4 Hz; PCH_2_), 26.28 (d, ^1^ *J*_CP_ = 13.4 Hz; PCH_2_), 26.02 (s; CH_2_), 24.4 (s; CH_3_CO), 24.2 (s; *C*H_3_CO), 23.2 (s; CH_2_), 23.0 (s; CH_2_), 22.9 ppm (s; *C*H_3_CO). ^31^P­{^1^H} NMR (162 MHz, CD_3_OD, 25 °C): δ = 51.7 (d, ^2^ *J*_PP_ = 36.58 Hz), 50.8 (d, ^2^ *J*_PP_ = 34.2 Hz), 50.3 (d, ^2^ *J*_PP_ = 35.0 Hz), 49.51 (d, ^2^ *J*_PP_ = 36.0 Hz). HRMS (ESI+) calcd for [C_74_H_69_N_4_O_4_P_4_^102^Ru_2_–OAc]^2+^: 673.1111; found, 673.1110. Elemental analysis for C_76_H_72_N_4_O_6_P_4_Ru: Calcd: C, 62.37; H, 4.96; N, 3.83. Found: C, 62.64; H, 5.16; N, 3.95.

### 4.6 Formic Acid Dehydrogenation

The desired Ru complex (2.86 μmol) was added to a 15 mL Schlenk flask, and the system was purged by applying 5 argon/vacuum cycles, followed by the addition of FA and pre-established amount of Et_3_N (0–100 mol % in relation to the FA). The mixture was stirred for enough time until the reaction stopped. The produced gas (CO_2_ and H_2_) was bubbled in a trap containing a saturated solution of NaOH to capture the CO_2_. The H_2_ was then collected using a graduated cylinder of 1 L, and the amount of produced gas was measured by the displacement of a water column. TOF_20_ and TOF_50_ were calculated by dividing the moles of H_2_ produced per mole of catalyst per hour at 20 and 50% yield, respectively. The catalyst recycling was performed by keeping the remaining catalyst, after completion of the reaction, in a Schlenk flask together with the residual triethylamine. Subsequent catalytic tests were carried out by adding additional FA to the Schlenk flask under an inert Ar atmosphere.

### 4.7 Transfer Hydrogenation of CO_2_

The 13 μ mols of the desired precatalyst were added to a 300 mL Parr reactor (4842) equipped with a stirrer, thermostatic bath, and manometer. The air was removed by applying three CO_2_/vacuum cycles, followed by the addition of 25 mL of a basic solution (0.5 mol L^–1^ of KOH) in H_2_O/*i*PrOH in different proportions. The system was pressurized to 50 bar, and the temperature was increased to 100 °C. The reaction mixture was maintained under stirring for 22 h. The product was analyzed by ^1^H NMR in D_2_O with isonicotinic acid as an internal standard.

## Supplementary Material



## Notes

Elemental analysis data; UV–vis, FTIR, NMR (^1^H, ^13^C, ^1^H–^1^H COSY, HMBC), and HRMS spectra of all complexes; cyclic voltammetry data; gas chromatography analysis of gases produced during formic acid dehydrogenation reactions; temperature effect on formic acid dehydrogenation; turnover number values for FADH; ^1^H NMR and two-dimensional ^1^H–^31^P HMBC spectra for mechanistic investigations of FADH; kinetic data and Arrhenius and Eyring plots for determination of thermodynamic parameters of complex **2**; FTIR and ^1^H NMR data for characterization of formic acid obtained by CO_2_ hydrogenation (PDF)

## References

[1] Ritchie, H. ; Rosado, P. ; Roser, M. CO_2_ emissions by fuel. Greenhouse gas emissions. In Our world in data, 2020.

[2] Onishi N., Iguchi M., Yang X., Kanega R., Kawanami H., Xu Q., Himeda Y. (2019). Development of effective catalysts for hydrogen storage
technology using formic acid. Adv. Energy Mater..

[3] Ferlin F., Valentine F., marrhocchi A., Vaccaro L. (2021). Catalytic biomass upgrading
exploiting liquid organic hydrogen carriers (LOHCs). ACS Sustain. Chem. Eng..

[4] Tang C., Fei S., Lin G. D., Liu Y. (2020). Natural liquid
organic hydrogen carrier
with low dehydrogenation energy: A first principles study. Int. J. Hydrogen Energy.

[5] Piccirilli L., Lobo Justo Pinheiro D., Nielsen M. (2020). Recent progress with pincer transition
metal catalysts for sustainability. Catalysts.

[6] Garron A., Epron F. (2005). Use of formic acid as reducing agent
for application in catalytic
reduction of nitrate in water. Water Res..

[7] Choi E. K., Park K. H., Lee H. B., Cho M., Ahn S. (2013). Formic acid
as an alternative reducing agent for the catalytic nitrate reduction
in aqueous media. Journal of Environ. Sci..

[8] Renz M. (2005). Ketonization
of carboxylic acids by decarboxylation: mechanism and scope. Eur. J. Org. Chem..

[9] Reutemann, W. K. ; Kieczka, H. Ullmann’s Encyclopedia of Industrial Chemistry; Wiley VCH, Weinheim, 2011.

[10] Chen Y., Yang Y., Liu X., Shi X., Wang C., Zhong H., Jin F. (2023). Sustainable production
of formic
acid and acetic acid from biomass. Mol. Catal..

[11] Park J. H., Lee D. W., Jin M. H., Lee Y. J., Song G. S., Park S. J., Jung H. J., Oh K. K., Choi Y. C. (2021). Biomass-formic
acid-hydrogen conversion process with improved sustainability and
formic acid yield: Combination of citric acid and mechanocatalytic
depolymerization. Chem. Eng. J..

[12] Achour M., Álvarez-Hernández D., Ruiz-López E., Megías-Sayago C., Ammari F., Ivanova S., Centeno M. A. ´. (2023). Formic acid as renewable reagent
and product in biomass upgrading. Tetrahedron
Green Chem..

[13] Xing R., Qi W., Huber G. W. (2011). Production
of furfural and carboxylic acids from waste
aqueous hemicellulose solutions from the pulp and paper and cellulosic
ethanol industries. Energy Environ. Sci..

[14] Jin F., Enomoto H. (2011). Rapid and highly selective
conversion of biomass into
value-added products in hydrothermal conditions: chemistry of acid/base-catalysed
and oxidation reactions. Energy Environ. Sci..

[15] Hafeez S., Harkou E., Spanou A., Al-Salem S. M., Villa A., Dimitratos N., Manos G., Constantinou A. (2022). Review on
recent progress and reactor set-ups for hydrogen production from formic
acid decomposition. Mater. Today Chem..

[16] Onishi N., Kanega R., Kawanami H., Himeda Y. (2022). Recent progress
in
homogeneous catalytic dehydrogenation of formic acid. Molecules.

[17] Loges B., Boddien A., Junge H., Beller M. (2008). Controlled generation
of hydrogen from formic acid amine adducts at room temperature and
application in H2/O2 fuel cells. Angew. Chem.,
Int. Ed..

[18] Sahoo P. K., Zhang Y., Das S. (2021). CO2-promoted reactions: an emerging
concept for the synthesis of fine chemicals and pharmaceuticals. ACS Catal..

[19] Zhang Y., Zhang T., Das S. (2020). Catalytic transformation
of CO 2
into C1 chemicals using hydrosilanes as a reducing agent. Green Chem..

[20] Sanz S., Benítez M., Peris E. (2010). A new approach to the reduction of
carbon dioxide: CO2 reduction to formate by transfer hydrogenation
in i PrOH. Organomet.

[21] Yin X., Moss J. R. (1999). Recent developments in the activation of carbon dioxide
by metal complexes. Coord. Chem. Rev..

[22] Masson G. H. C., Ballico M., Goi B. E., de Carvalho-Jr V. P., Baratta W. (2024). Light-enhancing ketone transfer hydrogenation
catalyzed
by diphosphine phenanthroline ruthenium complexes. Mol. Catal..

[23] Ballico M., Alessi D., Aneggi E., Busato M., Zuccaccia D., Allegri L., Damante G., Jandl C., Baratta W. (2024). Cyclometalated
and NNN Terpyridine Ruthenium Photocatalysts and Their Cytotoxic Activity. Molecules.

[24] Alessi D., Del Mestre P., Aneggi E., Ballico M., Beltrami A. P., Busato M., Cesselli D., Heidecker A. A., Zuccaccia D., Baratta W. (2023). Cyclometalated Ĉ N diphosphine
ruthenium catalysts for Oppenauer-type oxidation/transfer hydrogenation
reactions and cytotoxic activity. Catal. Sci.
Technol..

[25] Manbeck G.
F., Brewer K. J. (2013). Photoinitiated
electron collection in polyazine chromophores
coupled to water reduction catalysts for solar H2 production. Coord. Chem. Rev..

[26] Boddien A., Loges B., Junge H., Beller M. (2008). Hydrogen generation
at ambient conditions: application in fuel cells. ChemSusChem..

[27] Guan C., Zhang D. D., Pan Y., Iguchi M., Ajitha M. J., Hu J., Li H., Yao C., Huang M. H., Min S. (2017). Dehydrogenation of formic
acid catalyzed by a ruthenium complex with
an N, N′-diimine ligand. Inorg. Chem..

[28] Mphephu R., Joseph M. C., Swarts A. J. (2025). Pyrazolyl-Pyridine
Ruthenium (II)
Catalysts for Selective Hydrogen Generation Through Formic Acid Dehydrogenation. Eur. J. Inorg. Chem..

[29] Fidalgo J., Ruiz-Castañeda M., García-Herbosa G., Carbayo A., Jalón F. A., Rodríguez A. M., Manzano B. R., Espino G. (2018). Versatile Rh-and Ir-based catalysts
for CO2 hydrogenation, formic acid dehydrogenation, and transfer hydrogenation
of quinolines. Inorg. Chem..

[30] Mphuti T., Mphephu R., Joseph M., Swarts A. J. (2025). Unveiling Solvent-Dependent
Divergent Hydrogen Production Pathways during the Dehydrogenation
of Formic Acid Using N, N′-Iminopyridine Ruthenium (II) Complexes. Eur. J. Inorg. Chem..

[31] Schicktanz S. T., Steele W. I., Blaisdell A. C. (1940). Analysis of mixtures of aliphatic
acids. Ind. Eng. Chem., Anal. Ed..

[32] Narita K., Sekiya M. (1977). Vapor-liquid equilibrium
for formic acid-triethylamine
system examined by the use of a modified still. Formic acid-trialkylamine
azeotropes. Chem. Pharm. Bull..

[33] Bogado A. L., Paschai Darian L. K., Bürgy D., Dos Santos L. D. S., Ueno L. T. (2024). Hydrogen Production
by the Ruthenium (II) Complex Bearing
a Bulky PNP Ligand: A Catalyst for the Decomposition of Formic Acid
and/or Ammonium Formate. ACS Omega.

[34] Patra S., Awasthi M. K., Rai R. K., Deka H., Mobin S. M., Singh S. K. (2019). Dehydrogenation
of Formic Acid Catalyzed by Water-Soluble
Ruthenium Complexes: X-ray Crystal Structure of a Diruthenium Complex. Eur. J. Inorg. Chem..

[35] Iguchi M., Zhong H., Himeda Y., Kawanami H. (2017). Kinetic Studies on
Formic Acid Dehydrogenation Catalyzed by an Iridium Complex towards
Insights into the Catalytic Mechanism of High-Pressure Hydrogen Gas
Production. Chem.Eur. J..

[36] Vatsa A., Mishra A., Padhi S. K. (2022). Monitoring of catalytic
dehydrogenation
of formic acid by a ruthenium (II) complex through manometry. Inorg. Chem. Commun..

[37] Ballico M., Alessi D., Jandl C., Lovison D., Baratta W. (2022). Terpyridine
diphosphine ruthenium complexes as efficient photocatalysts for the
transfer hydrogenation of carbonyl compounds. Chem. Eur J..

[38] Baratta W., Chelucci G., Gladiali S., Siega K., Toniutti M., Zanette M., Zangrando E., Rigo P. (2005). Ruthenium (ii) Terdentate
CNN Complexes: Superlative Catalysts for the Hydrogen-Transfer Reduction
of Ketones by Reversible Insertion of a Carbonyl Group into the Ru-H
Bond. Angew. Chem., Int. Ed..

[39] Sanz S., Azua A., Peris E. (2010). (η6-arene) Ru
(bis-NHC)’complexes
for the reduction of CO2 to formate with hydrogen and by transfer
hydrogenation with iPrOH. Dalton Trans..

[40] Sanz S., Benítez M., Peris E. (2010). A new approach to the reduction of
carbon dioxide: CO2 reduction to formate by transfer hydrogenation
in iPrOH. Organomet.

[41] Feng X., Li J., Yang Z. (2022). Substituent’s Effects of PNP Ligands in Ru (II)-Catalyzed
CO2 Hydrogenation to Formate: Theoretical Analysis Considering Steric
Hindrance and Promotion of Hydrogen Bonding. Catalysts.

[42] Hey D. A., Sauer M. J., Fischer P. J., Esslinger E. M. H., Kühn F. E., Baratta W. (2020). Acetate acetylacetonate
ampy ruthenium (II) complexes as efficient catalysts for ketone transfer
hydrogenation. ChemCatChem.

[43] Wu J. Z., Li L., Zeng T. X., Ji L. N., Zhou J. Y., Luo T., Li R. H. (1997). Synthesis, characterization
and luminiscent DNA-binding study of
a series of ruthenium complexes containing 2-arylimidazo [f] 1, 10-phenanthroline. Polyhedron.

[44] Cai Z. B., Liu L. F., Zhou M. (2013). Synthesis of nickel
(II) complexes
containing modified phenanthroline ligands for potential nonlinear
optical applications. Opt. Mater..

[45] Frisch, M. J. ; Trucks, G. W. ; Schlegel, H. B. ; Scuseria, G. E. ; Robb, M. A. ; Cheeseman, J. R. ; Scalmani, G. ; Barone, V. ; Petersson, G. A. ; Nakatsuji, H. ; Li, X. ; Caricato, M. ; Marenich, A. V. ; Bloino, J. ; Janesko, B. G. ; Gomperts, R. ; Mennucci, B. ; Hratchian, H. P. ; Ortiz, J. V. ; Izmaylov, A. F. ; Sonnenberg, J. L. ; Williams-Young, D. ; Ding, F. ; Lipparini, F. ; Egidi, F. ; Goings, J. ; Peng, B. ; Petrone, A. ; Henderson, T. ; Ranasinghe, D. ; Zakrzewski, V. G. ; Gao, J. ; Rega, N. ; Zheng, G. ; Liang, W. ; Hada, M. ; Ehara, M. ; Toyota, K. ; Fukuda, R. ; Hasegawa, J. ; Ishida, M. ; Nakajima, T. ; Honda, Y. ; Kitao, O. ; Nakai, H. ; Vreven, T. ; Throssell, K. ; Montgomery, J. A., Jr. ; Peralta, J. E. ; Ogliaro, F. ; Bearpark, M. J. ; Heyd, J. J. ; Brothers, E. N. ; Kudin, K. N. ; Staroverov, V. N. ; Keith, T. A. ; Kobayashi, R. ; Normand, J. ; Raghavachari, K. ; Rendell, A. P. ; Burant, J. C. ; Iyengar, S. S. ; Tomasi, J. ; Cossi, M. ; Millam, J. M. ; Klene, M. ; Adamo, C. ; Cammi, R. ; Ochterski, J. W. ; Martin, R. L. ; Morokuma, K. ; Farkas, O. ; Foresman, J. B. ; Fox, D. J. Gaussian 16; Revision C.02, Gaussian, Inc.: Wallingford CT, 2019.

[46] Adamo C., Barone V. (1999). Toward reliable density functional methods without
adjustable parameters: The PBE0 model. J. Chem.
Phys..

[47] a Dunning, T. H., Jr. ; Hay, P. J. . In Modern Theoretical Chemistry; SchaeferIII, H. F. , Ed.; Plenum: New York, 1977; Vol. 3, pp 1–28.

[48] Weigend D. F., Ahlrichs R. (2005). Balanced basis sets of split valence, triple zeta valence
and quadruple zeta valence quality for H to Rn: Design and assessment
of accuracy. Phys. Chem. Chem. Phys..

