# Supplementary material for: Molecular Hydrogen Production from Formic Acid by Cationic Phenanthroline Ruthenium Complexes: Experimental and DFT Mechanistic Insights
Source: ACS Omega. 2026 Jan 21;11(4):5947–58. doi: 10.1021/acsomega.5c10186 (PMC12878708; doi:10.1021/acsomega.5c10186)
Supplement: Supplementary file 1 [file ao5c10186_si_001.pdf]

Molecular Hydrogen Production from Formic Acid by Cationic Phenanthroline Ruthenium Complexes: Experimental and DFT Mechanistic Insights

Gustavo H. C. Masson [a,b,c], Douglas H. N. Santos [a,b], Lucas S. Santos [d], André L. Bogado [d], Leonardo T. Ueno [e], Beatriz E. Goi [a], Walter Baratta [b*], Valdemiro P. Carvalho-Jr [a*]

[a]*Faculdade de Ciências e Tecnologia (FCT) da Universidade Estadual Paulista (UNESP), Presidente Prudente, SP, 19060-900, Brazil*

[b]*Dipartimento di Scienze AgroAlimentari, Ambientali e Animali (DI4A) – Università di Udine Via Cotonificio108, 33100 Udine, Italy*

[c]*Universidade Estadual de Campinas (UNICAMP), Instituto de Química, 13083-970, Campinas, SP, Brazil*

[d] *Universidade Federal de Uberlândia, Instituto de Ciências Exatas e Naturais do Pontal, ICENP–UFU, 38304-402, Ituiutaba, MG, Brazil*

[e]*Instituto Tecnológico de Aeronáutica (ITA), Comando-Geral de Tecnologia Aeroespacial, Departamento de Química, São José dos Campos, São Paulo 12228-900, Brazil*

*Correspondence to: valdemiro.carvalho@unesp.br and walter.baratta@uniud.it

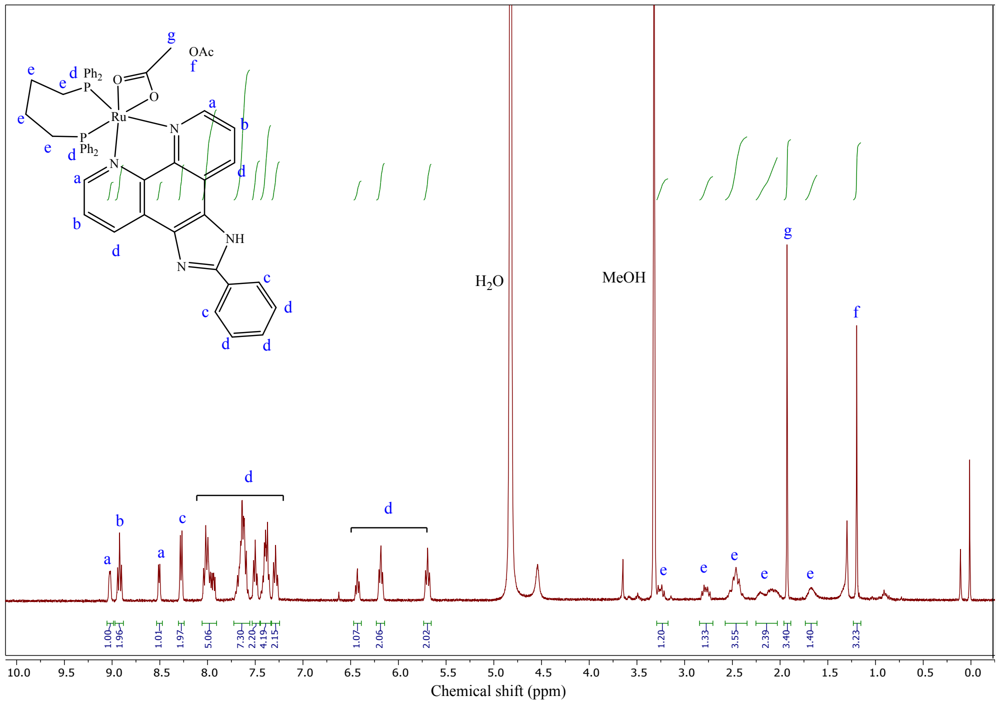

Figure S1. ^{1}H NMR spectrum of **3** in CD_3OD (δ in ppm).

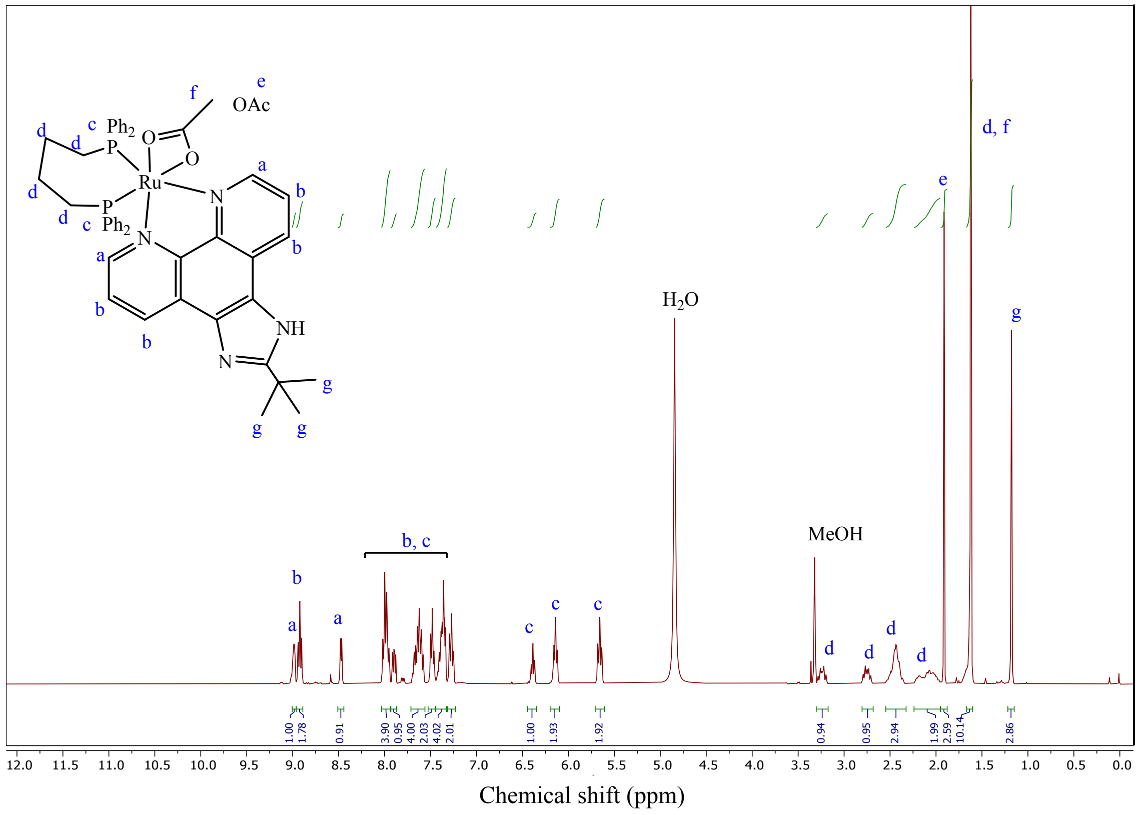

Figure S2. ^{1}H NMR spectrum of **4** in CD_3OD (δ in ppm).

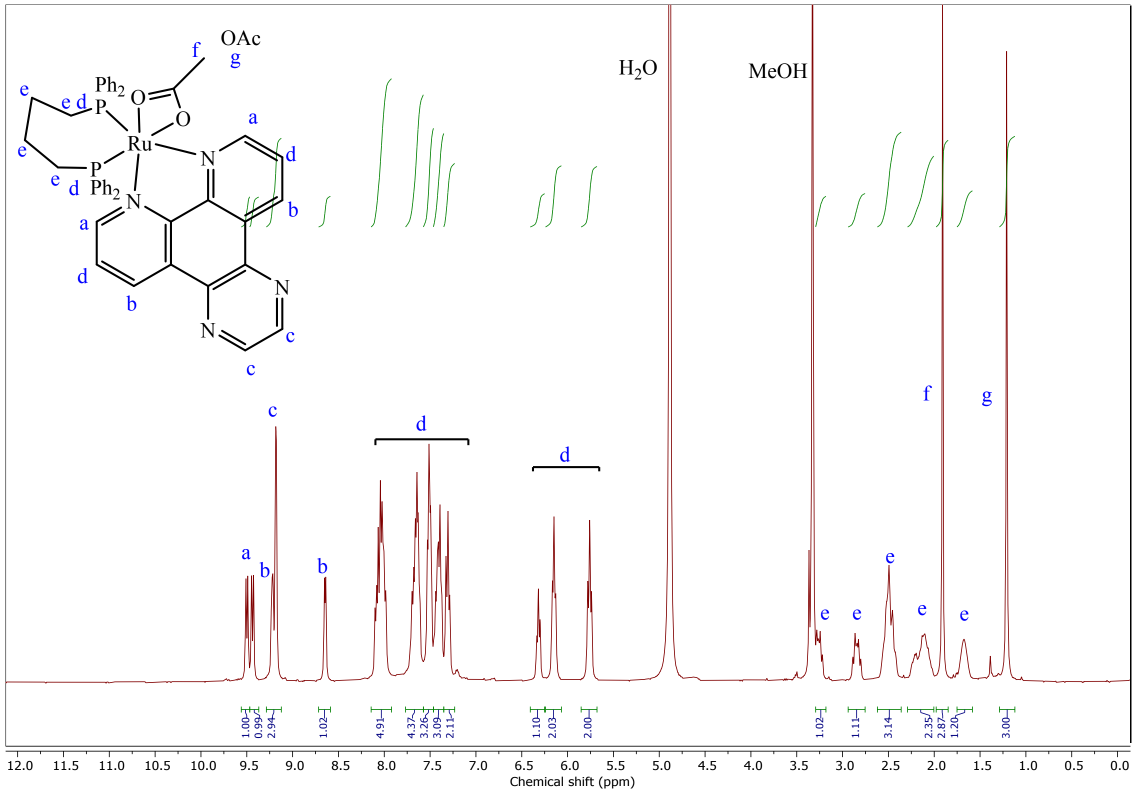

Figure S3. ^{1}H NMR spectrum of **5** in CD_3OD (δ in ppm).

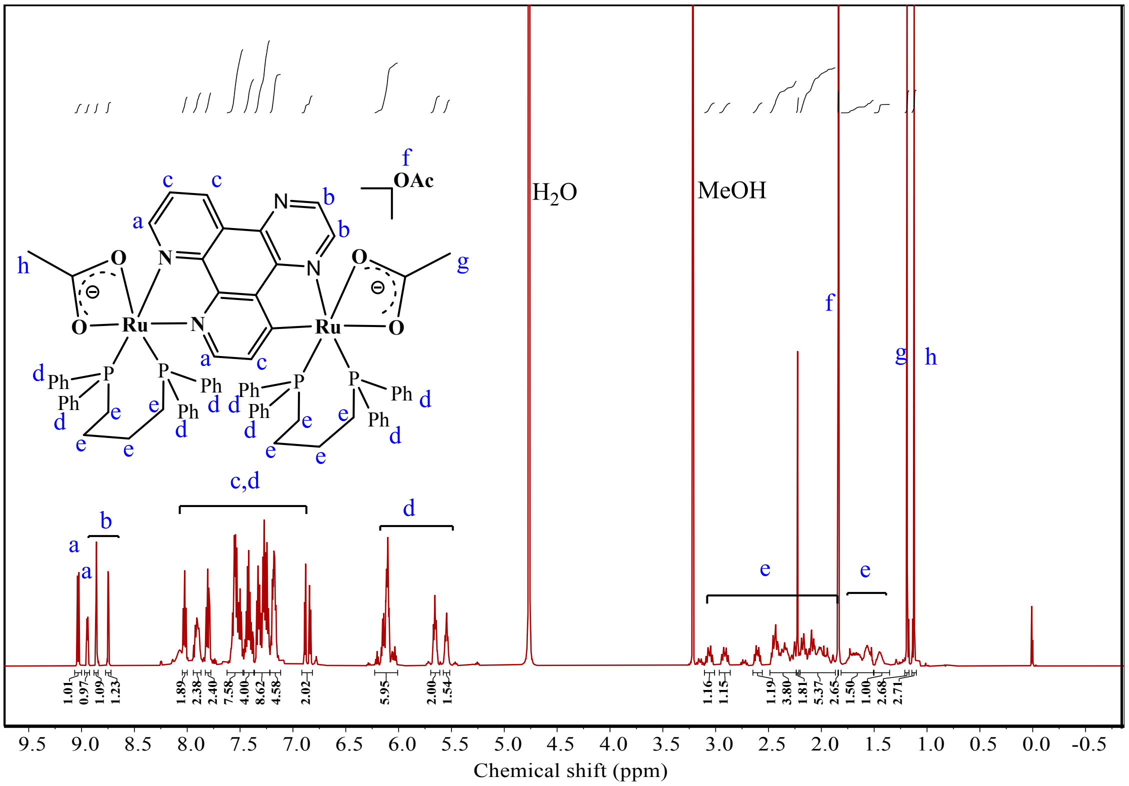

Figure S4. ^{1}H NMR spectrum of **7** in CD_3OD (δ in ppm).

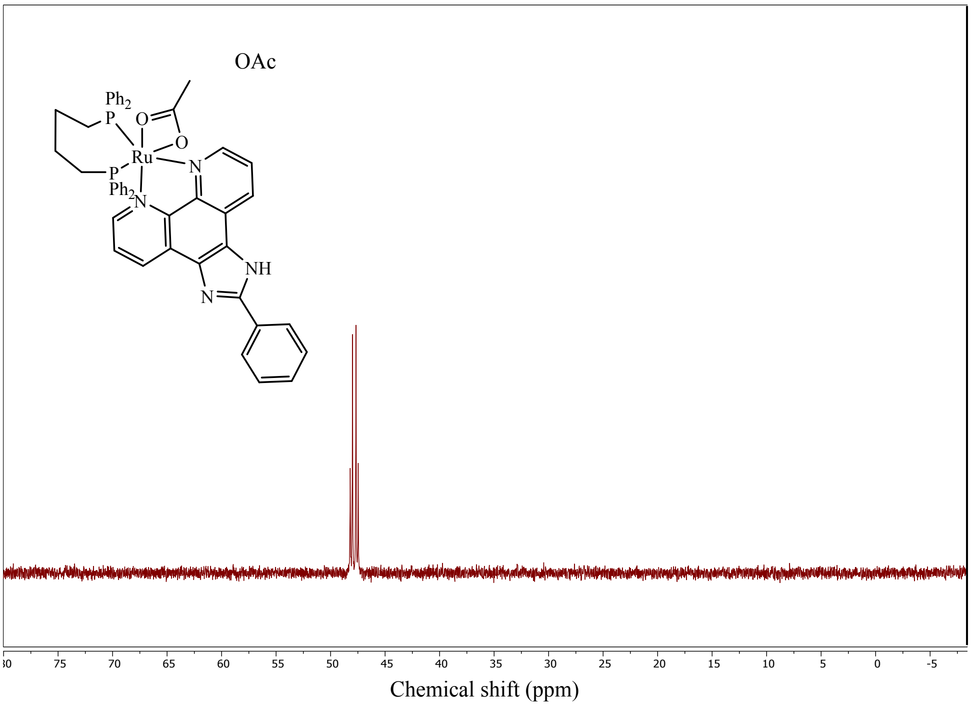

Figure S5. $^{31}P\{^{1}H\}$ NMR spectrum of **3** in CD_3OD (δ in ppm).

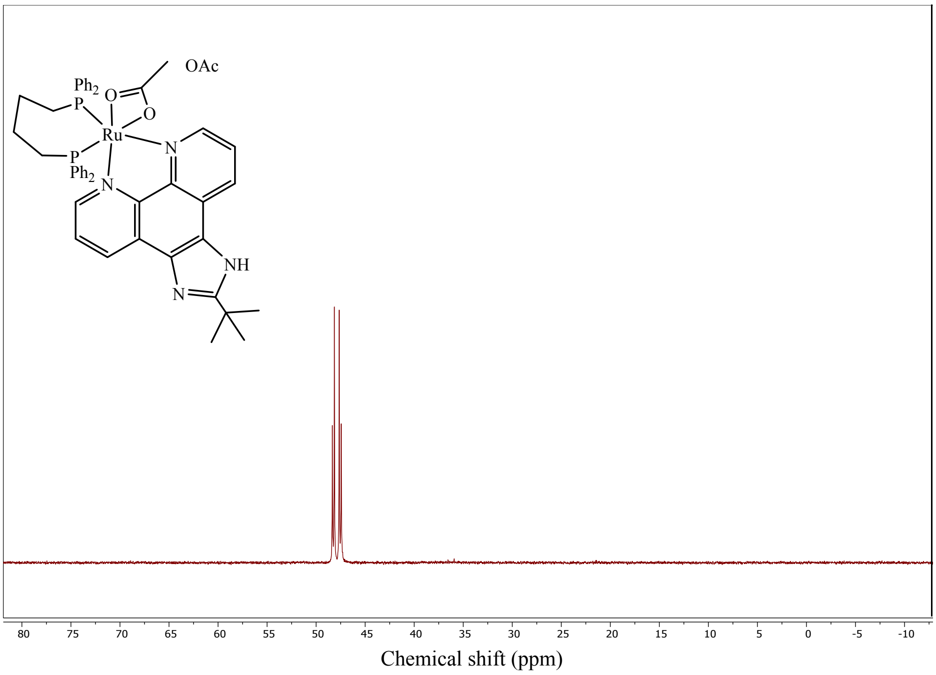

Figure S6. $^{31}P\{^{1}H\}$ NMR spectrum of **4** in CD_3OD (δ in ppm).

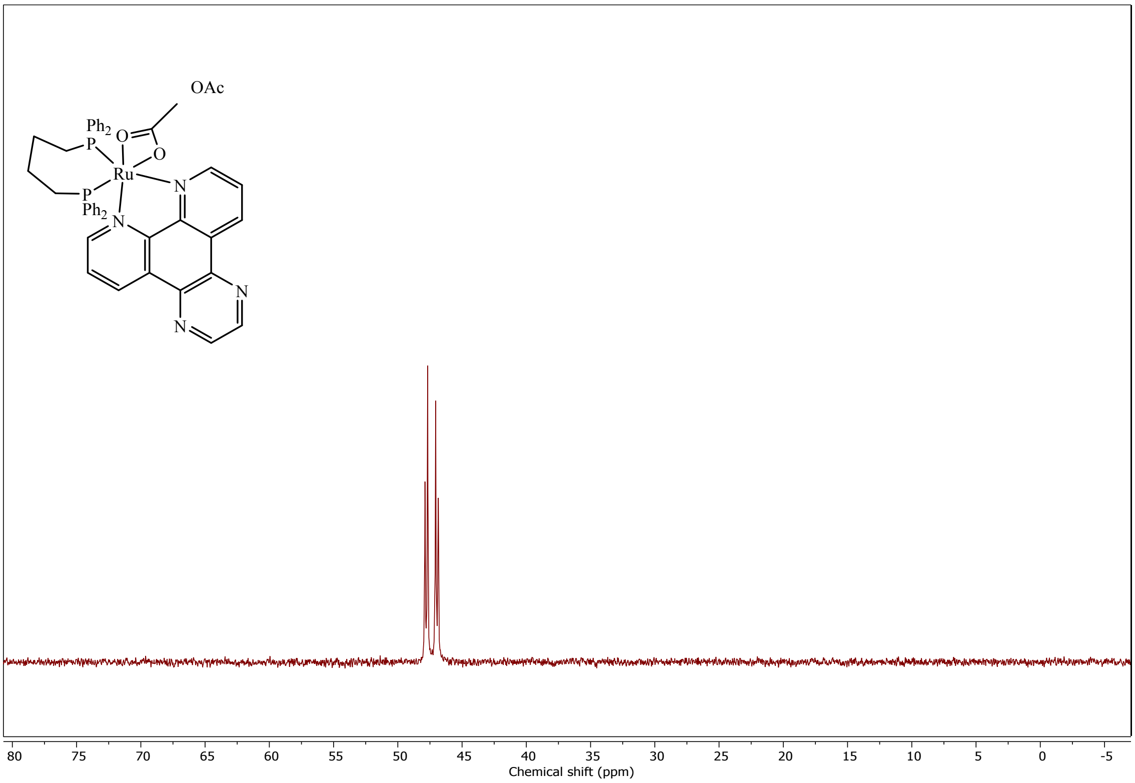

Figuere S7. $^{31}P\{^1H\}$ NMR spectrum of **5** in CD_3OD (δ in ppm).

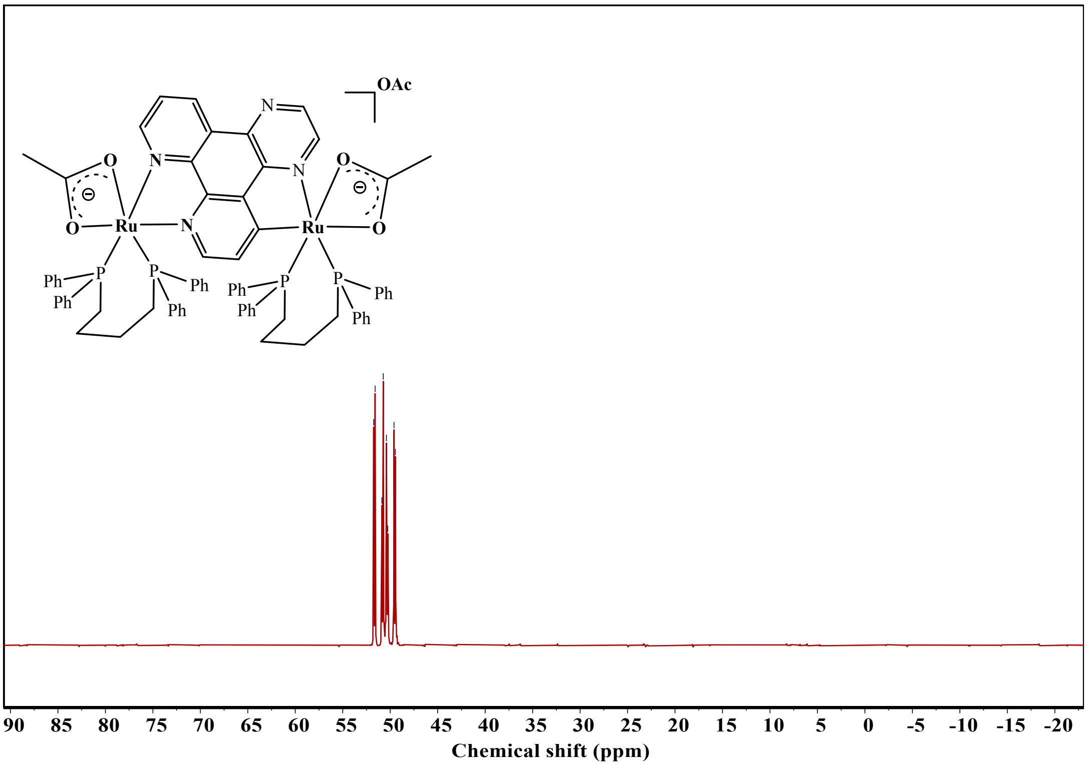

Figure S8. $^{31}P\{^1H\}$ NMR spectrum of **7** in CD_3OD (δ in ppm).

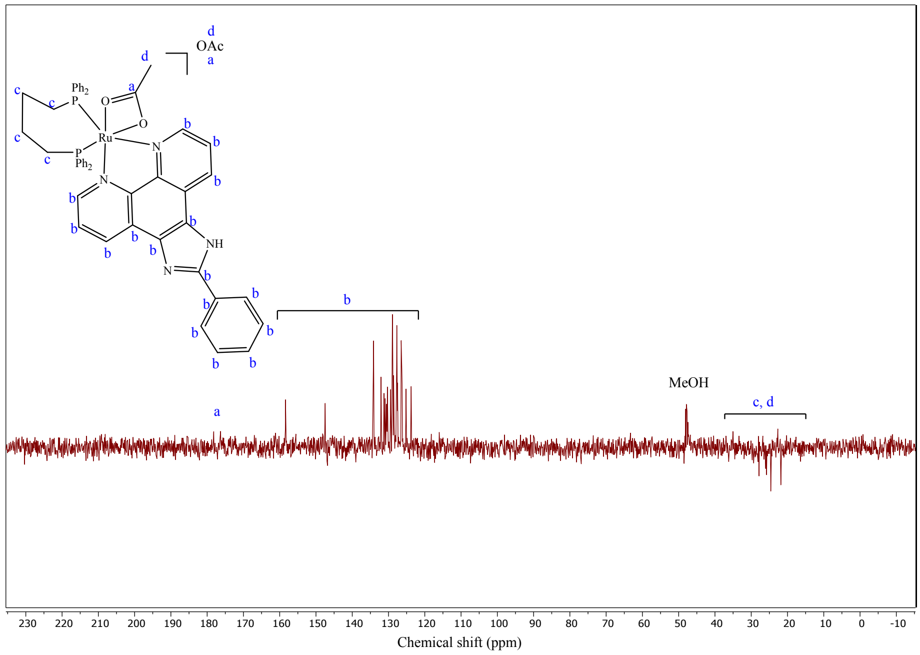

Figure S9. DEPTQ-135 $^{13}C\{^1H\}$ spectrum of **3** in CD_3OD (δ in ppm).

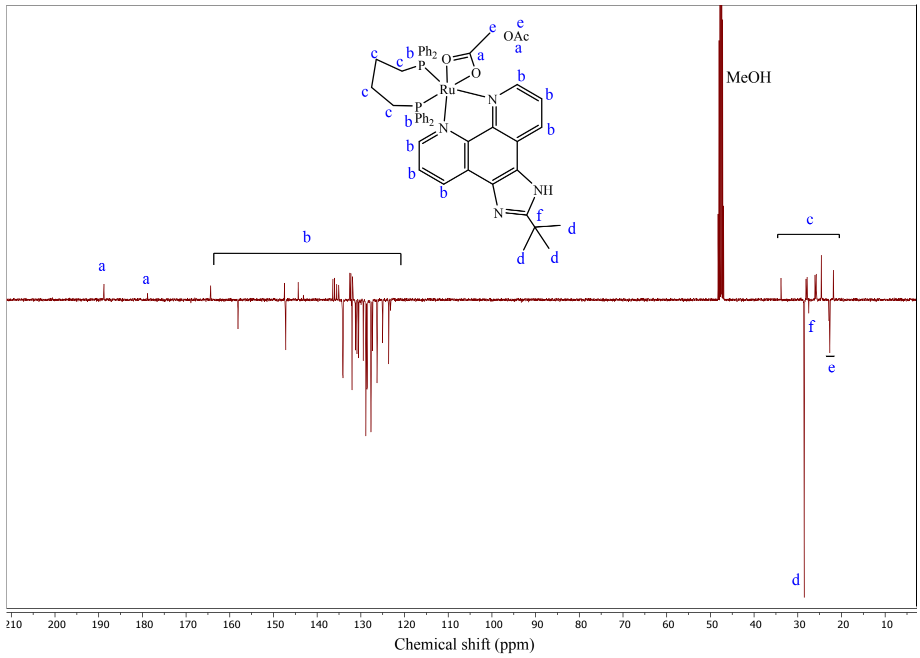

Figure S10. DEPTQ-135 $^{13}C\{^1H\}$ spectrum of **4** in CD_3OD (δ in ppm).

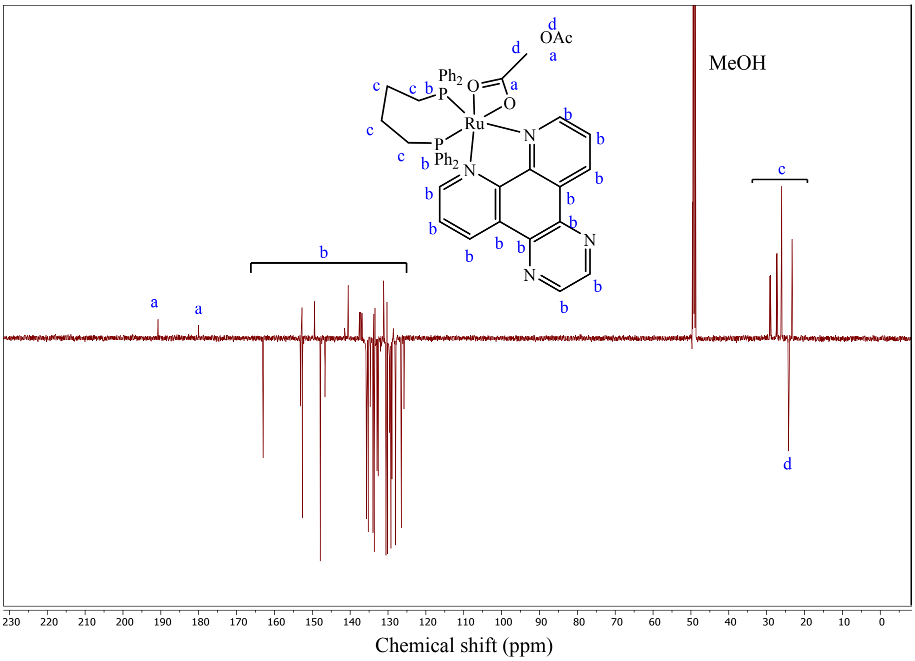

Figure S11. DEPTQ-135 $^{13}C\{^{1}H\}$ spectrum of **5** in CD_3OD (δ in ppm).

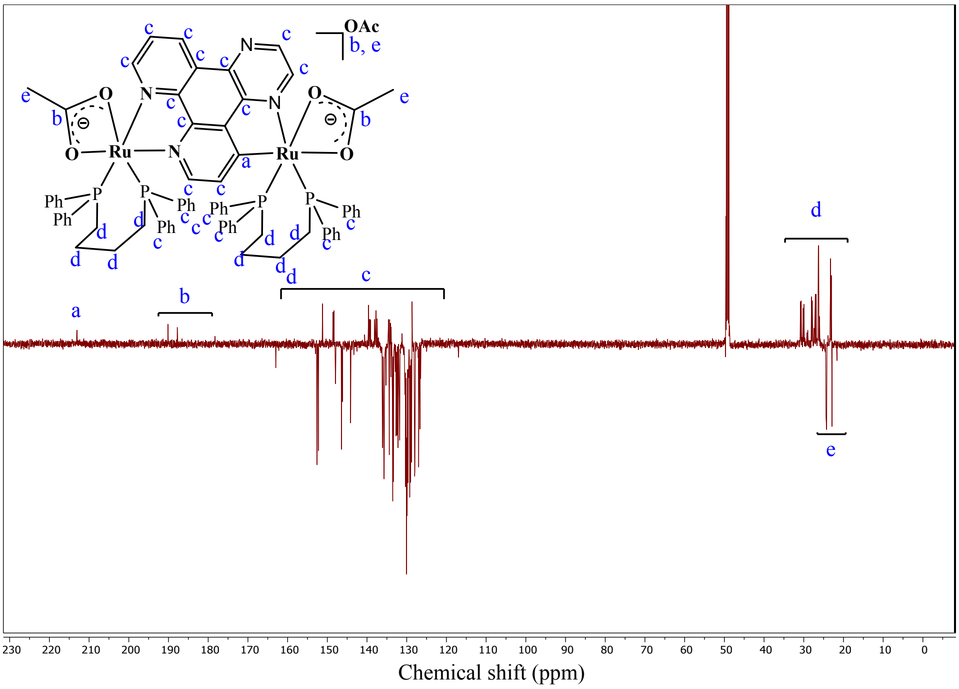

Figure S12. DEPTQ-135 $^{13}C\{^{1}H\}$ spectrum of **7** in CD_3OD (δ in ppm).

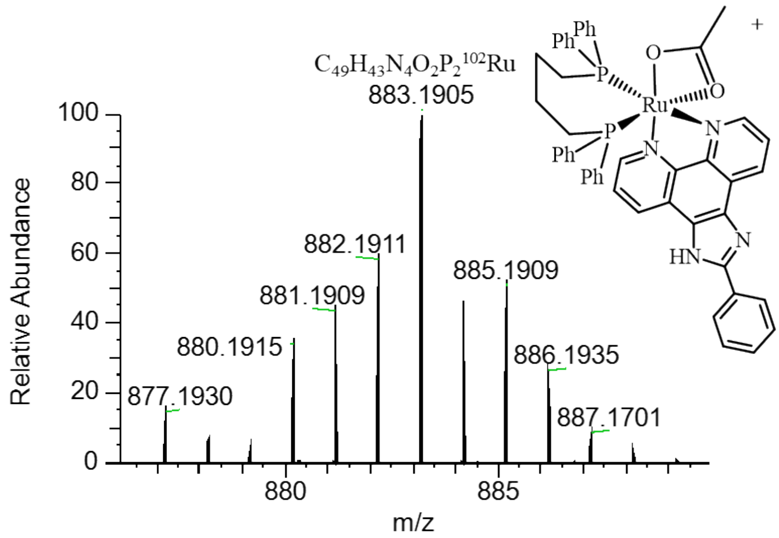

Figure S13. HRMS of complex **3** in positive mode using methanol as solvent.

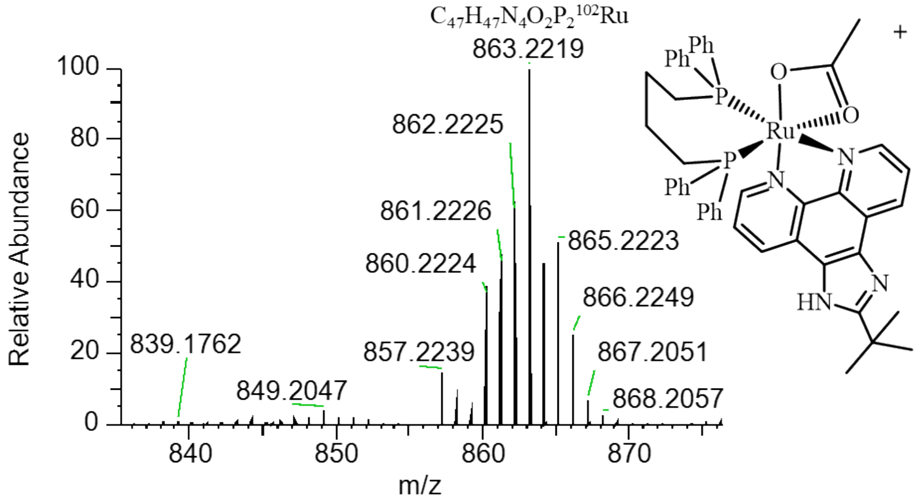

Figure S14. HRMS of complex **4** in positive mode using methanol as solvent.

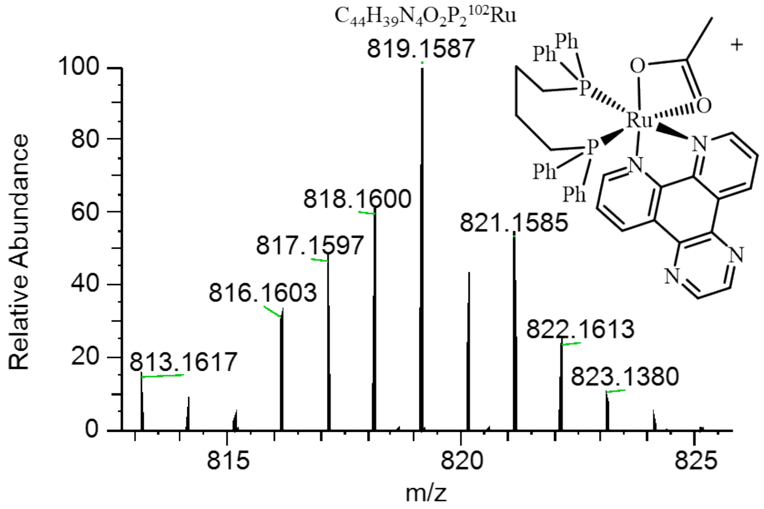

Figure S15. HRMS of complex **5** in positive mode using methanol as solvent.

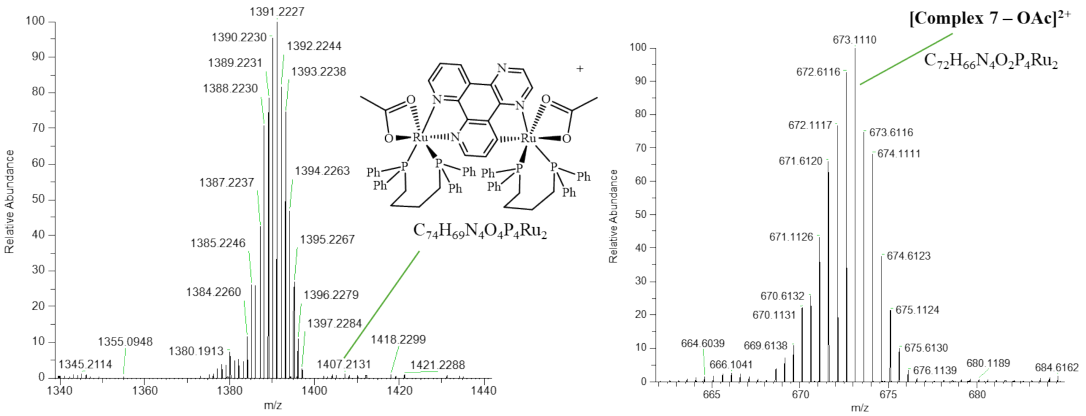

Figure S16. HRMS of complex **7** in positive mode using methanol as solvent.

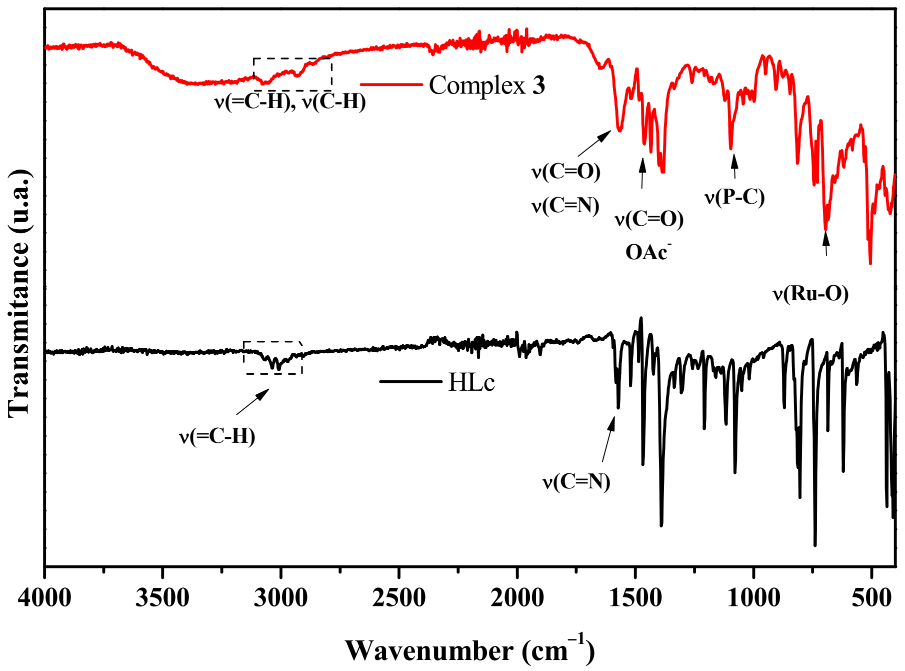

Figure S17. FTIR spectra of complex **3** and its respective ligand.

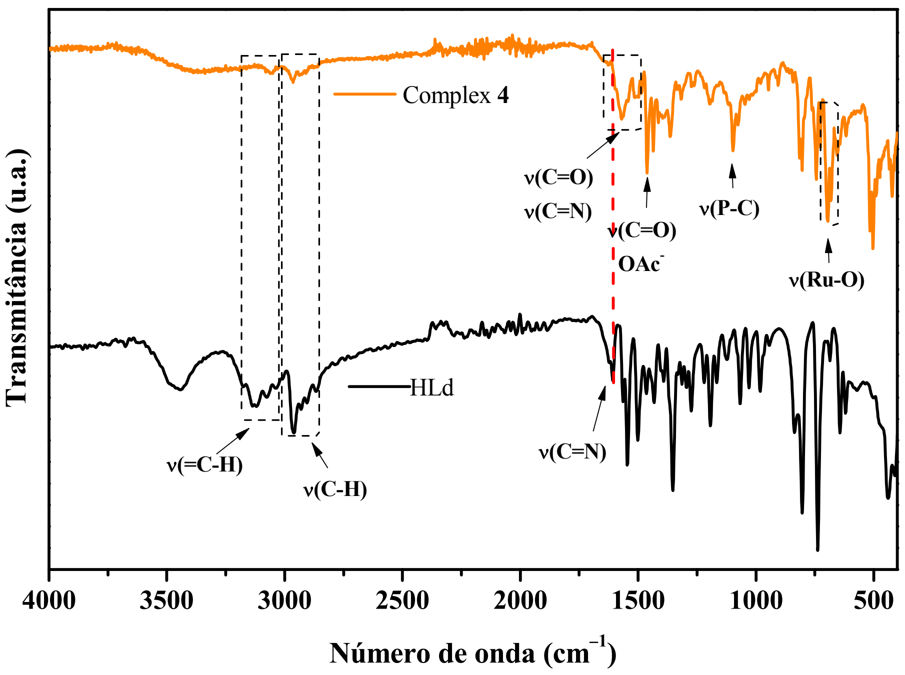

Figure S18. FTIR spectra of complex **4** and its respective ligand.

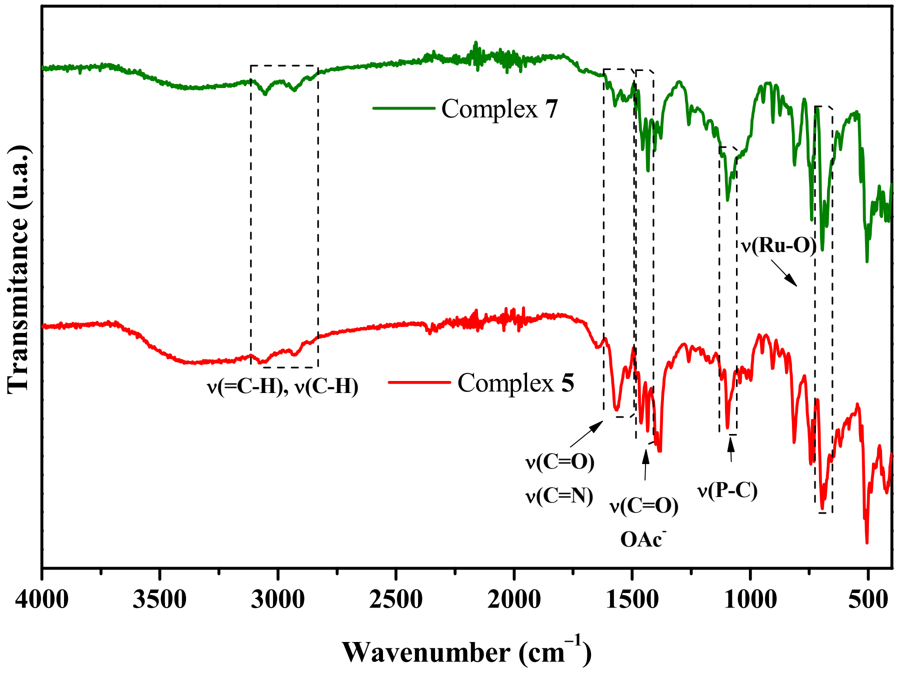

Figure S19. FTIR spectra of complex **5** and **7**.

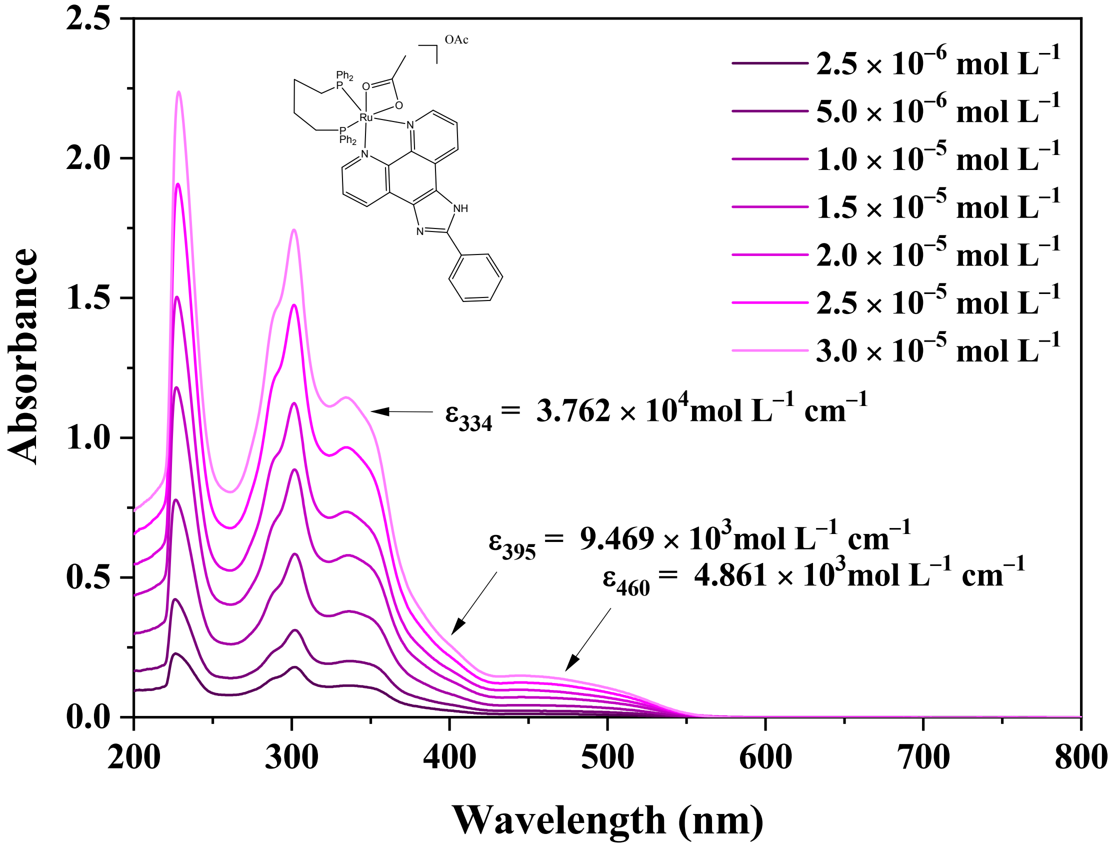

Figure S20. Absorption spectra of complex **3** in CH_2Cl_2 at 25 ºC in different [Ru] concentrations.

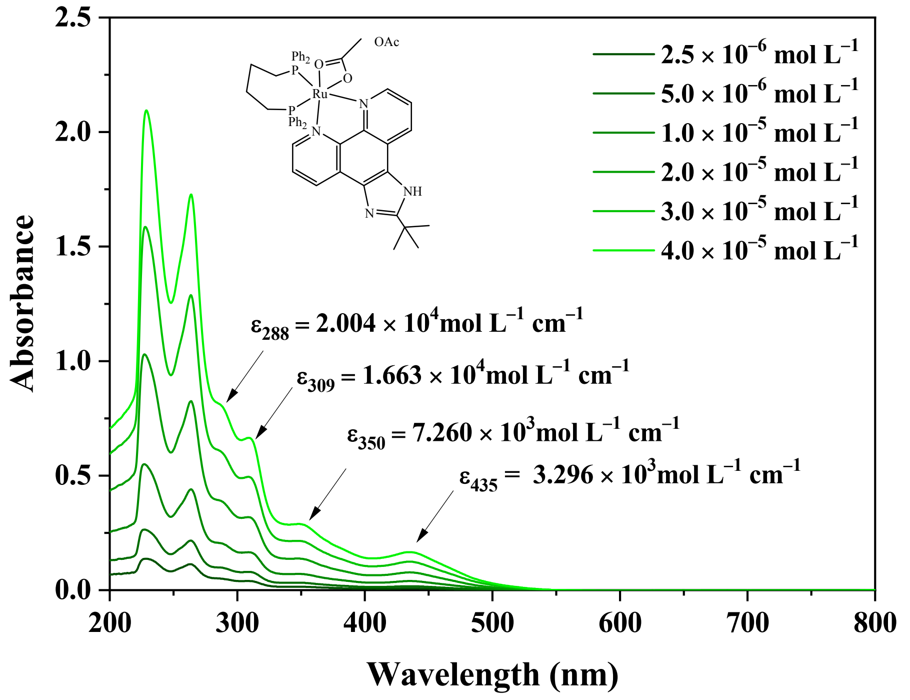

Figure S21. Absorption spectra of complex **4** in CH_2Cl_2 at 25 ºC in different [Ru] concentrations.

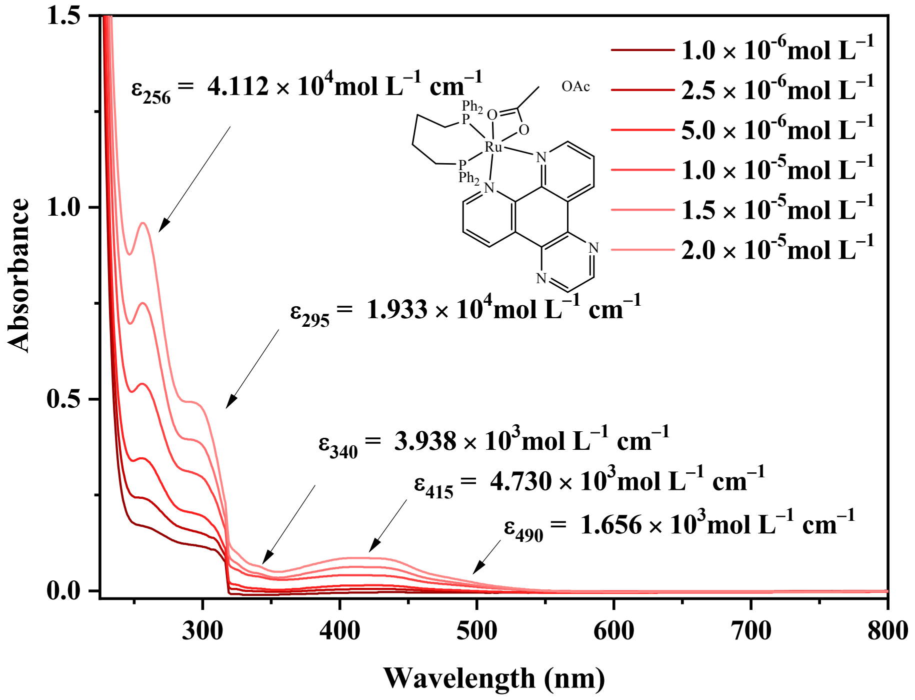

Figure S22. Absorption spectra of complex **5** in CH_2Cl_2 at 25 ºC in different [Ru] concentrations.

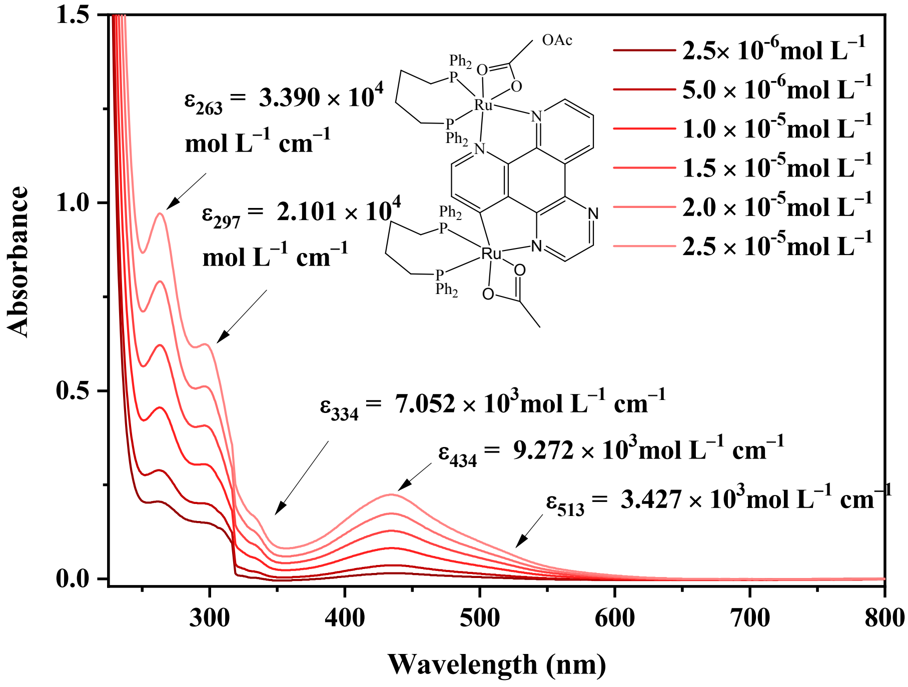

Figure S23. Absorption spectra of complex **7** in CH_2Cl_2 at 25 ºC in different [Ru] concentrations.

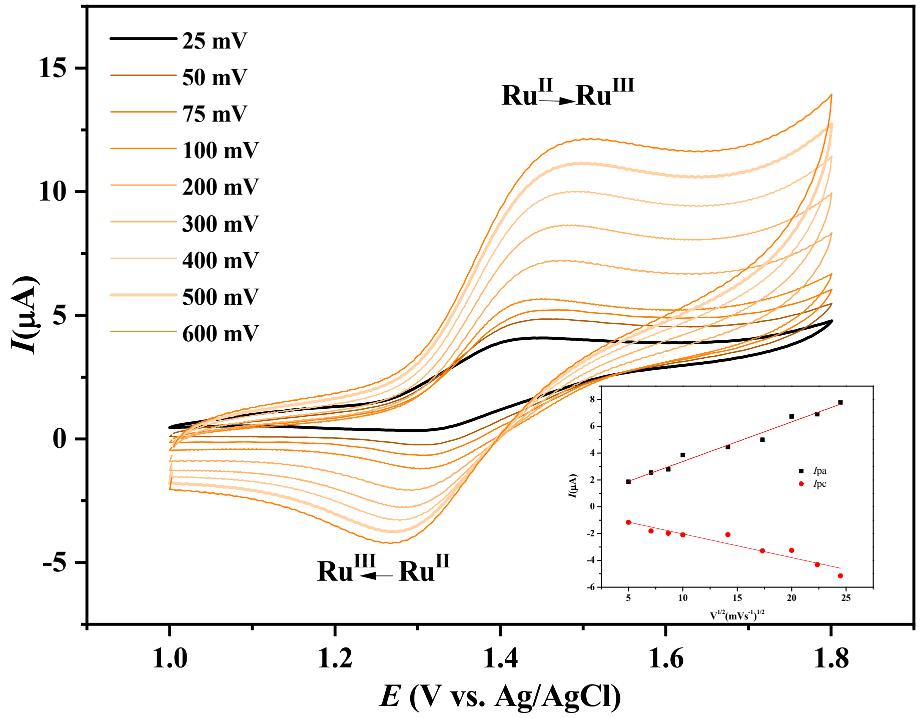

Figure S24. Cyclic voltammograms of complex **3** in CH_2Cl_2 at 25 °C. Scanning anodically at scan rate of 100 mV s^{-1}. [Ru] = 1.0 × 10^3 mol L^{-1}; [n-Bu_4NPF_6] = 0.1 mol L^{-1}.

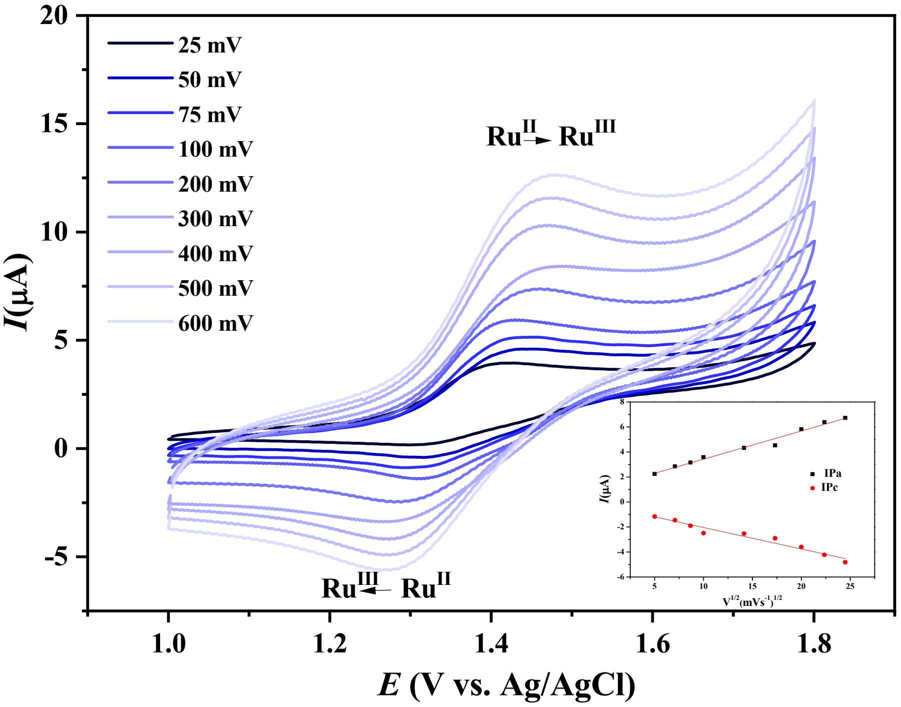

Figure S25. Cyclic voltammograms of complex **4** in CH_2Cl_2 at 25 °C. Scanning anodically at scan rate of 100 mV s^{-1}. [Ru] = 1.0 × 10^3 mol L^{-1}; [n-Bu_4NPF_6] = 0.1 mol L^{-1}.

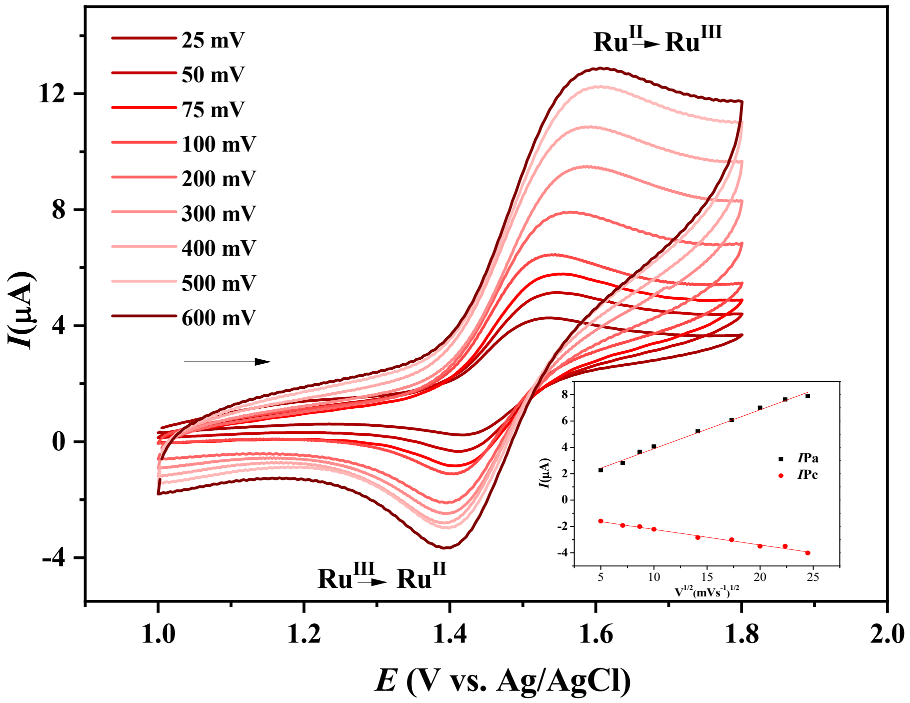

Figure S26. Cyclic voltammograms of complex **5** in CH_2Cl_2 at 25 °C. Scanning anodically at scan rate of 100 mV s^{-1}. [Ru] = 1.0 × 10^3 mol L^{-1}; [n-Bu_4NPF_6] = 0.1 mol L^{-1}.

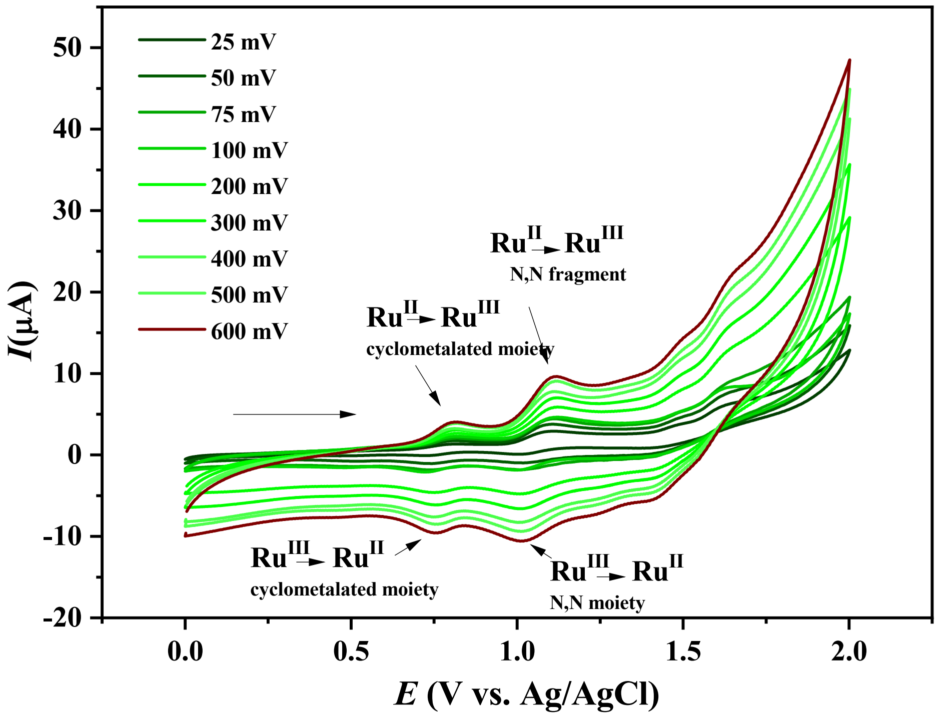

Figure S27. Cyclic voltammograms of complex **7** in CH_2Cl_2 at 25 °C. Scanning anodically at scan rate of 100 mV s^{-1}. [Ru] = 1.0 × 10^3 mol L^{-1}; [n-Bu_4NPF_6] = 0.1 mol L^{-1}.

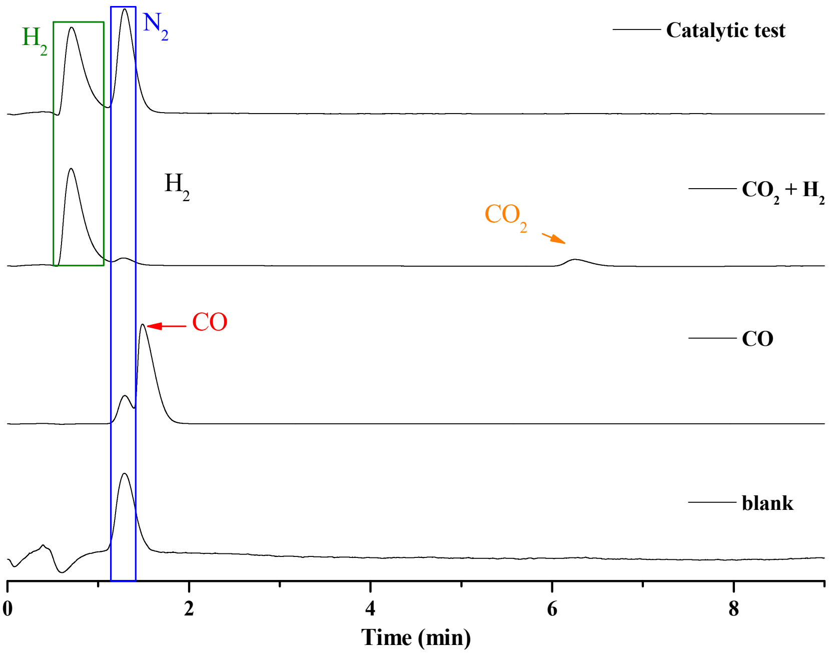

Figure S28. Gas chromatography of the gas produced in the dehydrogenation reactions of FA using complex **7**. Conditions: Et_3N/AF = 75 mol%, Cat/AF = 1000, t = 90 ºC.

Table S1. Effect of the temperature on the FADH with **6** in toluene.[a]

Entry	T (ºC)	Yield (%)	TOF_{20} (h^{-1})[b]	t (min)[c]
1	90	93	2727	4.4
2	100	100	3529	3.4
3	105	100	5217	2.3
4	110	100	9230	1.3

[a]Reactions conducted using FA/Cat = 1000 and 75 mol% of Et_3N. [b]TOF_{20} at 20% of yield. [c]Time for 20% of yield.

Table S2. TON values for FADH using complexes **1-7**.[a]

Entry	Cat.	TON
1	**1**	210
2	**2**	290
3	**3**	70
4	**4**	430
5	**5**	640
6	**6**	930
7	**7**	930

[a]TON for 1 cycle of FADH using complexes 1-7. Conditions: FA/Cat = 1000 and 75 mol% of Et_3N. [b]TOF_{20} at 20% of yield.

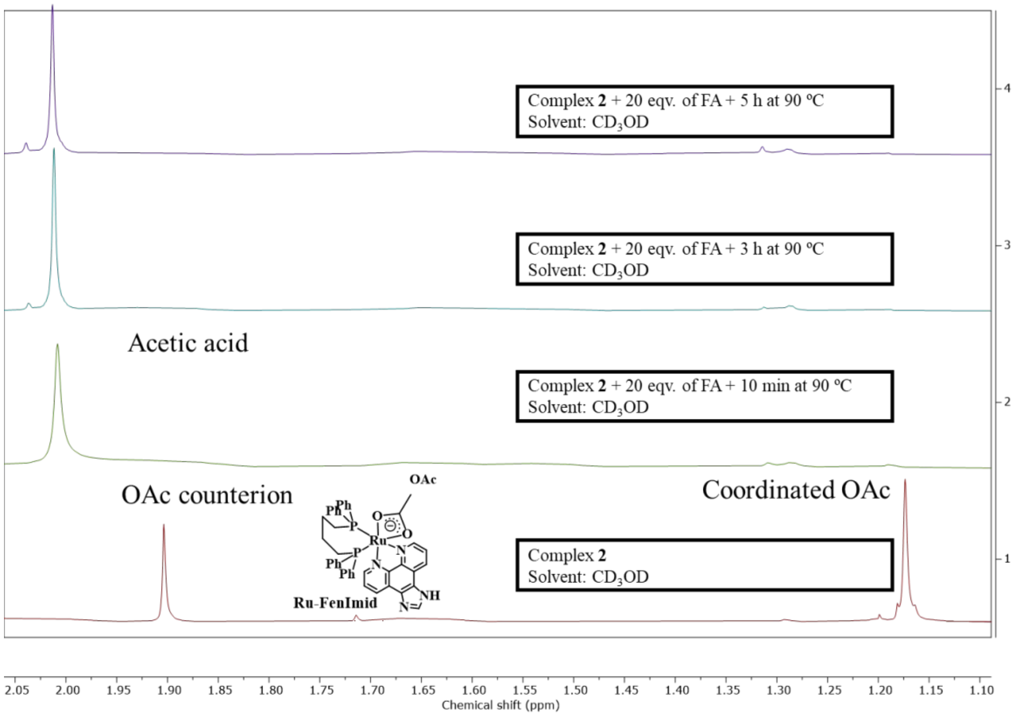

Figure 29. ^{1}H NMR monitoring in CD_3OD of the counterion exchange of complex **2** by the addition of formic acid – (δ in ppm).

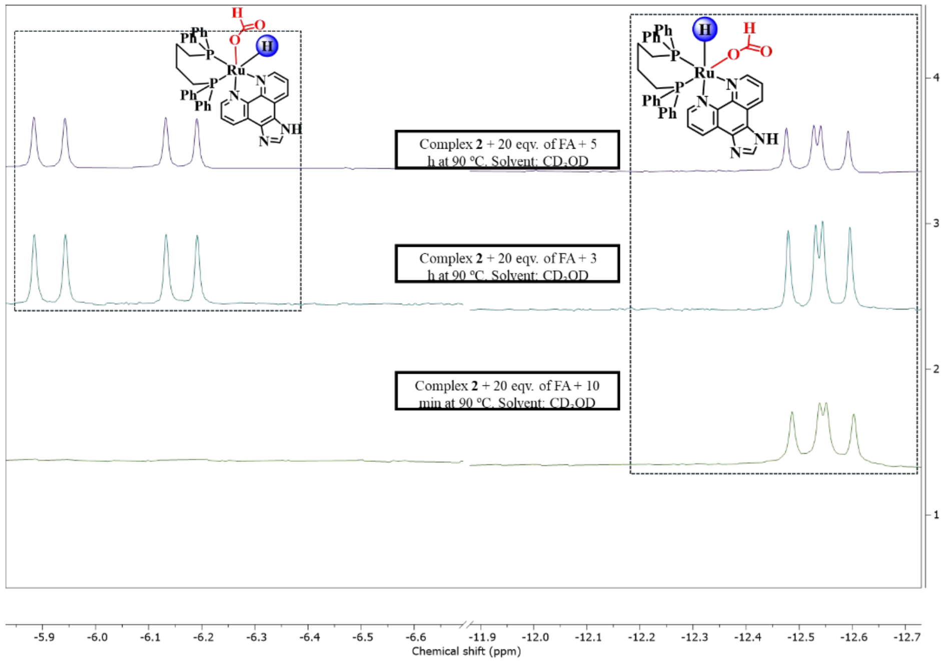

Figure S30. Monitoring by ^{1}H NMR in CD_3OD of the formation of Ru-H in the axial and equatorial position from **2** by the addition of formic acid and exposure to a temperature of 90 ºC – (δ in ppm).

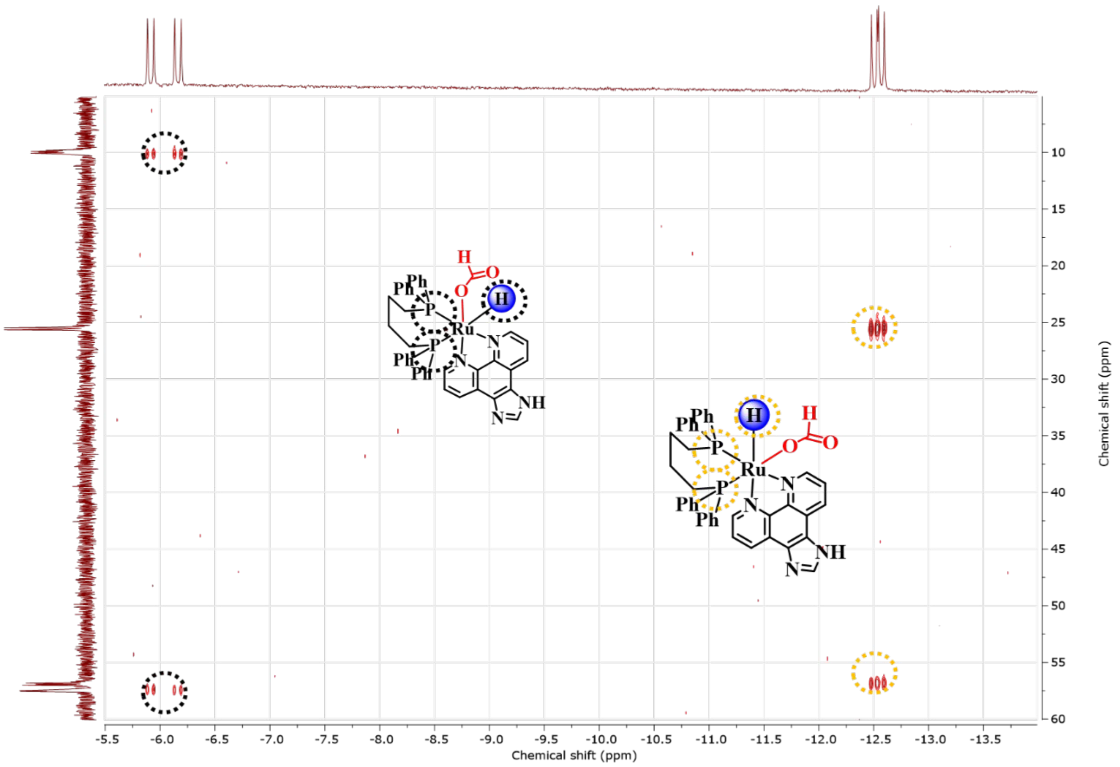

Figure S31. Bidimensional ^{1}H-^{31}P NMR (HMBC) spectrum of the Ru-H compound from **2**, obtained after the addition of 20 equivalents of FA in $CD^{3}OD$ and heated at 90 °C for 5 hours.

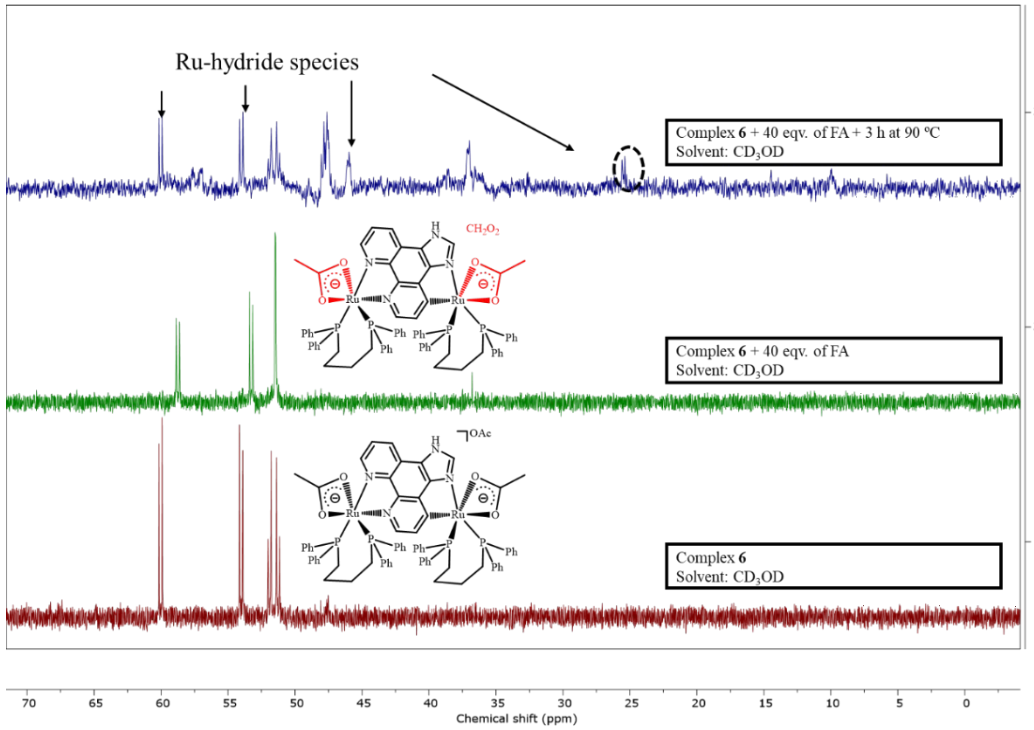

Figure S32. $^{31}P\{^{1}H\}$ NMR monitoring in CD_3OD of the formation of Ru-hydride species from complex **2** using formic acid at 90 °C – (δ in ppm).

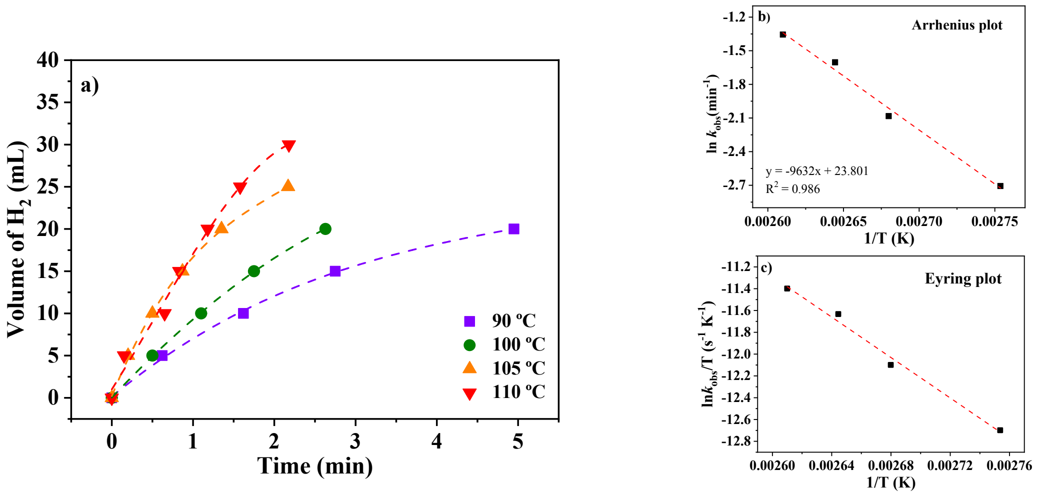

Figure S33. (a) FADH using **2** in toluene at different temperatures. (b) E_a determination using Arrhenius plot. (c) ΔS‡ and ΔH‡ determination using Eyring plot.

Table S3. Thermodynamic parameters for complexes **2** and **6**.

Complex	E_a (kJ mol^{-1})	ΔH‡ (kJ mol^{-1})	ΔS‡ (J mol^{-1} K^{-1})	ΔG‡ (kJ mol^{-1})[a]
**2**	80.1	77.1	−90.9	117.0
**6**	60.3	57.2	−149.6	113.0

[a] ΔG‡ at 100 °C.

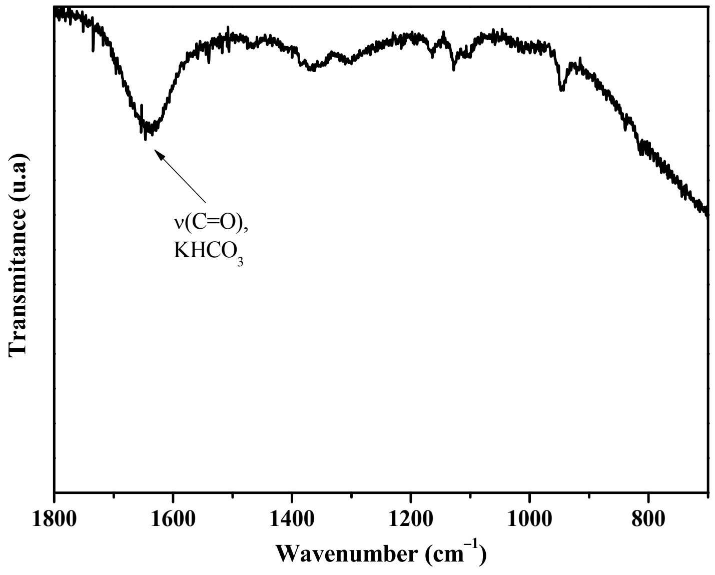

Figure S34. FTIR of the product obtained from the reaction of TH to CO_2 in the absence of catalyst.

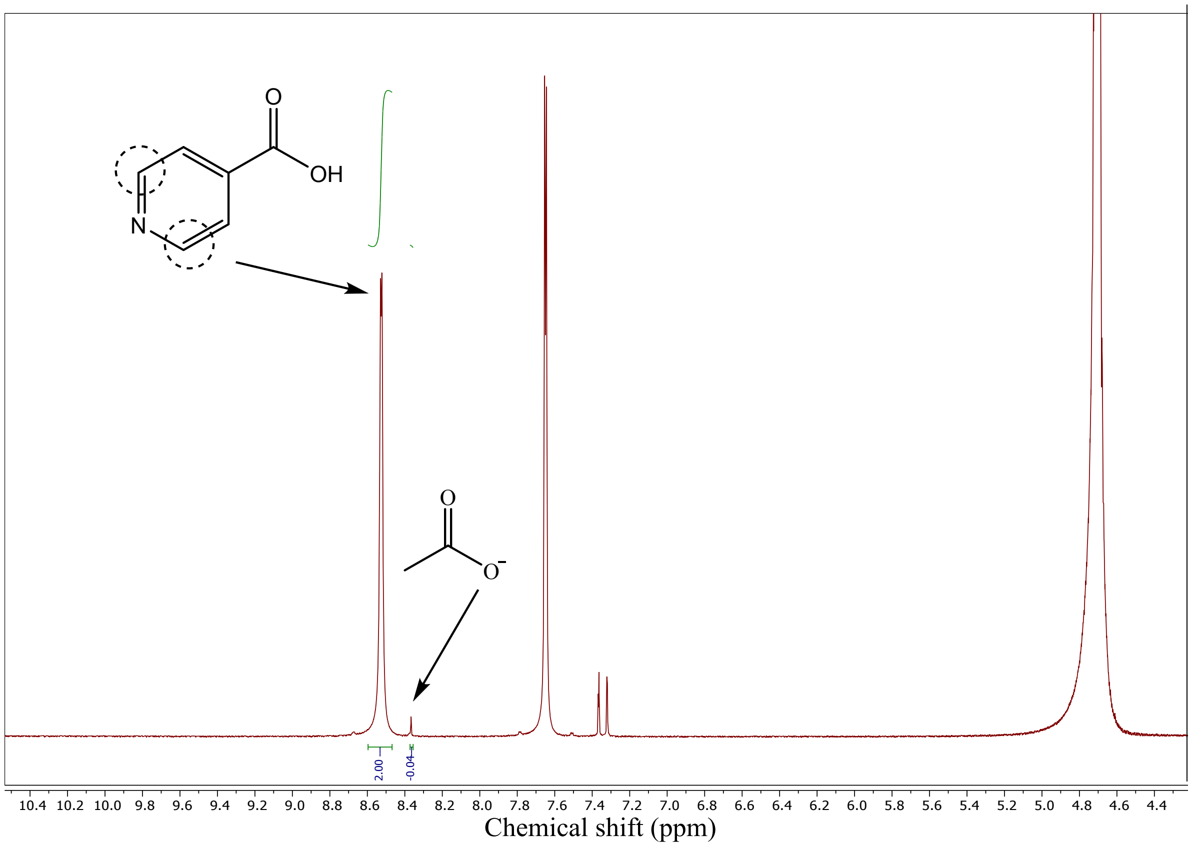

Figure S35. ^{1}H NMR of potassium formate obtained in the transfer hydrogenation reaction to CO_2 using the complex **1**.
